# Metabolomic Analyses of *Leishmania* Reveal Multiple Species Differences and Large Differences in Amino Acid Metabolism

**DOI:** 10.1371/journal.pone.0136891

**Published:** 2015-09-14

**Authors:** Gareth D. Westrop, Roderick A. M. Williams, Lijie Wang, Tong Zhang, David G. Watson, Ana Marta Silva, Graham H. Coombs

**Affiliations:** 1 Strathclyde Institute of Pharmacy and Biomedical Sciences, University of Strathclyde, Glasgow, United Kingdom; 2 Institute of Biomedical and Environmental Health Research, University of the West of Scotland, Paisley, United Kingdom; 3 Instituto de Biologia Molecular e Celular, Universidade do Porto, Porto, Portugal; National Center for Cell Science, INDIA

## Abstract

Comparative genomic analyses of *Leishmania* species have revealed relatively minor heterogeneity amongst recognised housekeeping genes and yet the species cause distinct infections and pathogenesis in their mammalian hosts. To gain greater information on the biochemical variation between species, and insights into possible metabolic mechanisms underpinning visceral and cutaneous leishmaniasis, we have undertaken in this study a comparative analysis of the metabolomes of promastigotes of *L*. *donovani*, *L*. *major* and *L*. *mexicana*. The analysis revealed 64 metabolites with confirmed identity differing 3-fold or more between the cell extracts of species, with 161 putatively identified metabolites differing similarly. Analysis of the media from cultures revealed an at least 3-fold difference in use or excretion of 43 metabolites of confirmed identity and 87 putatively identified metabolites that differed to a similar extent. Strikingly large differences were detected in their extent of amino acid use and metabolism, especially for tryptophan, aspartate, arginine and proline. Major pathways of tryptophan and arginine catabolism were shown to be to indole-3-lactate and arginic acid, respectively, which were excreted. The data presented provide clear evidence on the value of global metabolomic analyses in detecting species-specific metabolic features, thus application of this technology should be a major contributor to gaining greater understanding of how pathogens are adapted to infecting their hosts.

## Introduction

Leishmaniasis is a debilitating disease for approximately 12 million people living mainly within the tropics [1], caused by some 20 species of the protozoan parasite *Leishmania* [2]. *Leishmania* has a digenetic life cycle alternating between motile, extracellular promastigotes in the insect vector and non-motile, intracellular amastigotes in the mammalian host. The parasite replicates as procyclic promastigotes in the gut of phlebotomine sandflies, differentiating to non-replicating but infectious metacyclic promastigotes that are injected into the mammalian bloodstream when the insect feeds. The metacyclic promastigotes are taken up by mononuclear phagocytes where they differentiate into and replicate as amastigotes within a parasitophorous vacuole. The disease pathology is dependent upon the species of the infecting *Leishmania* with the so-called cutaneous, mucocutaneous and visceral leishmaniases resulting from intracellular infection with the amastigote form of the parasite of primarily macrophages in, respectively, superficial subcutaneous tissue, submucosal tissues of the nose and mouth, or internal organs including, in particular, the liver and spleen. The diseases caused are of differing severity, with visceral leishmaniasis being lethal if not treated. The pathogenicity of *Leishmania* is known to be mainly determined by the parasite species itself, although the aetiology underlying the different clinical outcomes is not fully understood and it is clear that the immune status of the host and co-infections with other pathogens such as HIV and tuberculosis can affect pathogenicity [2].

The major causative agent of visceral leishmaniasis is *L*. *donovani*, whilst two major agents responsible for cutaneous leishmaniasis are *L*. *major* and *L*. *mexicana*. It was anticipated that the differing abilities of various *Leishmania* species to survive in different host tissues and the resultant differing pathogenicity would be explained by species-specific genes. However, comparative analyses of the genomes of *L*. *major*, *L*. *braziliensis* and *L*. *infantum* [3] and *L*. *donovani* and *L*. *mexicana* [4,5,6] have revealed not only a strong conservation of gene content and synteny, but also rather few species-specific genes. Moreover, these species-specific genes have not so far provided the information necessary to decipher the different disease phenotypes [4]. Thus the phenotypic differences between the various species are now thought to be due to a differential regulation of gene expression and protein functions and not simply due to the possession of different genes. This suggests that a detailed analysis of transcriptomic and proteomic variations may help identify the mechanisms that underpin disease tropism, but also that analysis of the metabolomes, the net end products of gene transcription, of each parasite should provide key information on the web of complex metabolite interactions that are responsible for disease tropism and give some indication of genes which are differentially regulated in the different species.

Methods and technologies for the detection, identification and measurement with high specificity of the metabolites within a cell and its environment have improved immensely in recent years and have spawned the burgeoning field of ‘metabolomics’. They are now available to be applied in research on pathogens such as parasites and so contribute significantly to understanding their biology as well as enabling better drug discovery, disease diagnostics and treatment [7,8]. Several studies on the metabolomes of *Leishmania* have been reported to date, yielding both detailed methods that can be successful and insights into the biochemistry and mechanisms of drug resistance of individual species [9–14]. All have involved analysis of the promastigote forms of a single parasite species with only one study analysing the amastigote form at all; this reflects the lack of methods for obtaining amastigotes pure and in sufficient numbers for analysis of this type.

Logarithmic and stationary phase promastigotes from *in vitro* cultures showed distinct metabolic profiles, thought to reflect increased differentiation into metacyclic forms [10,11,13]. Differences included decreased levels of nucleosides, increased levels of nucleobases and apparent changes in membrane glycerol phospholopid structure predicted to lead to lower membrane fluidity. Logarithmic and stationary phase promastigotes have also been compared with amastigotes, albeit those obtained from *in vitro* cultures and known as axenic amastigotes, using *L*. *mexicana*. [13]. This study showed that promastigotes in culture consume more glucose and some amino acids than they need, releasing partially catabolised products into the medium. Analysis with C^13^-U-glucose revealed the release of C^13^-labeled CO_2_, succinate, alanine, acetyl-CoA and to a lesser extent aspartate, malate, glutamate and glutamine. Similarly, label from C^13^-U-glutamate was incorporated into the secreted metabolites CO_2_, succinate, alanine, acetyl-CoA and glutamine, with highest levels released by logarithmic phase promastigotes. In contrast, these metabolites were hardly secreted by axenic amastigotes. Indeed, the amastigotes were shown to have a slower metabolism and used glucose and amino acids sparingly, even when present at high concentration in the medium. This was referred to as the stringent metabolic response and is characterised by a decrease in uptake and catabolism of glucose and amino acids and an increase in fatty acid catabolism by β-oxidation. These authors also reported that amastigotes derived from lesions in animals have a similar metabolic profile to axenic amastigotes, thus indicating that the differences between axenic amastigotes and promastigotes should also reflect naturally-occurring differences. Metabolomic profiling of promastigotes has also been used to investigate the mode of action of antileishmanial drugs [9,11,12,14]; unfortunately it is not currently possible to investigate amastigotes, the disease causing stage, in the same way. Comparison of *L*. *donovani* strains with different susceptibility to sodium stibogluconate identified numerous metabolic differences including levels of intermediates in pathways linked to protection against oxidative stress (arginine metabolism for production of spermidine, transsulfuration pathway producing cysteine) and composition of glycerol phospholipids, resulting in higher membrane fluidity [9,11]. In contrast, metabolomic and lipidomic analysis of *L*. *infantum* promastigotes [12] showed that membrane phospholipids were not altered following treatment with miltefosine, although an increase in the turnover of internal membrane lipids was indicated by increases in alkane fragments.

These few previous metabolomic studies have involved different *Leishmania* species and varying approaches which make species comparison difficult. Thus the main aim of this current study was to use an untargeted metabolomics approach to gain information on the differences in the metabolic profiles of the three different species, with the hope that this may provide interesting insights into the innate biological variations and possibly enable hypotheses to be formulated concerning the adaptations of the individual species to their distinct niches within the mammalian host. We studied the lines of *L*. *donovani*, *L*. *major* and *L*. *mexicana* used for the benchmark genome analyses [3–5] and further developed our mass spectrometry-based metabolomics techniques to include confirmation of the identity of many of the metabolites by comparison with standards or by interpretation of MS^2^ spectra of significantly varying metabolites for which there were no standards. In addition, quantification of the levels of significant metabolites in the spent growth media was carried out. The enhanced methodologies developed have been used to compare the metabolomes of promastigotes at equivalent stages of *in vitro* growth and the changes in the media resulting from parasite growth.

## Materials and Methods

### Leishmania parasites

*L*. *donovani* (MHOM/NP/03/BPK282/0 clone 14), *L*. *major* (MHOM/IL/81/Friedlin) and *L*. *mexicana* (MHOM/GT/2001/U1103, clone 25) were used in this study. *L*. *donovani* promastigotes had been cloned from an isolate from a visceral leishmaniasis patient sensitive to pentavalent antimonials in Nepal, as descrifbed by Rijal and co-workers [15]. *L*. *donovani*, *L*. *major* and *L*. *mexicana* promastigotes were grown in modified Eagle’s medium (designated HOMEM medium, Invitrogen) supplemented with 10% (v/v) heat inactivated fetal calf serum (FCS, PAA Laboratories) and at 26°C. The same batch of serum was used for all cultures. Cultures were set up initially at a concentration of 2.5 x 10^5^ parasites/ml and all parasites were harvested for metabolite extraction after 3 or 6 days of *in vitro* growth; the growth progression of the cultures was monitored quantitatively by microscopic analysis.

### Chemicals and solvents

High performance liquid chromatography (HPLC) grade acetonitrile (ACN), chloroform and methanol were purchased from Fisher Scientific, UK. HPLC grade water was produced by a Direct-Q 3 Ultrapure Water System from Millipore, UK. AnalaR grade formic acid (98%) was obtained from BDH-Merck, UK. Ammonium carbonate, ammonium hydroxide solution (30–33%), all the standard compounds and the reagents and kits required for the various assays performed were purchased from Sigma-Aldrich, UK.

### Preparation of mixed metabolite standard solutions

Each metabolite standard was prepared at 1 mg/ml with HPLC grade methanol and water (1:1, v/v) and stored at -20°C. 100 μl was taken from each stock solution and about 45 metabolites were mixed and then made up to 10 ml with acetonitrile for each standard solution (see S1 Table for details of compositions). Consequently, the final concentration for each metabolite standard was 10 μg/ml with 185 metabolite standards being distributed into the four mixed metabolite standard solutions (detailed in S1 Table). In order to avoid identity confusion, isomers were distributed into different standard solutions and the presence of in-source fragments producing spurious metabolites was also carefully verified. These standards were run routinely to aid identification of sample metabolites. The names and formulae of these 185 metabolite standards are given in S1 Table. Additional metabolite standards were also used in this study for identification of the LC-MS (liquid chromatography mass spectrometry) features that showed clear species-specificity; they were prepared as described above and are detailed in S2 Table.

### *Leishmania* extracts for metabolite analysis

Promastigotes from four independently growing 10 ml cultures (biological replicates) were harvested after 6 days at 26°C and metabolite extraction was performed as previously described [16]. Briefly, the 10 ml cultures containing promastigotes were quenched in a dry ice/ethanol bath by rapid temperature decrease to 2°C and immediately transferred to ice. The samples were maintained at low temperature throughout the extraction procedure to prevent the interconversion of metabolic intermediates. Two aliquots of 4 x 10^7^ cells were taken from each culture flask (one for metabolite extraction and the other for protein estimation) and harvested by centrifugation at 1800 x g for 10 min at 4°C, the supernatant (designated the spent medium) was removed and stored on ice. Cell pellets were washed 2 times in 1 ml of phosphate buffered saline (PBS) at 4°C by centrifugation at 1800 x g for 10 min at 4°C. For cell disruption and metabolite extraction, cell pellets were resuspended in 200 μl cold chloroform/methanol/water (20/60/20, v/v/v) and incubated 1 h in a Thermomixer (14,000 rpm, 4°C). Proteins precipitated by the organic solvent in the extraction buffer were removed by centrifugation at 12,000 g for 10 min at 4°C. The supernatant containing the extracted metabolites was then recovered and stored at -70°C until analysed. To 75 μl of spent medium was added 300 μl of cold chloroform/methanol (20/60, v/v) followed by incubation for 1 h in a Thermomixer (14,000 rpm, 4°C). After centrifugation at 12,000 g for 10 min at 4°C, the supernatant was recovered and stored at -70°C until analysed.

### Metabolic profiling by LC-MS

The metabolic profiling was performed on an Accela 600 HPLC system combined with an Exactive (Orbitrap) mass spectrometer from Thermo Fisher Scientific (Bremen, Germany). The LC conditions used for ZIC-HILIC (Hydrophilic Interaction Liquid Chromatography) were the same as in our previous studies [16] and for ZIC-pHILIC were as follows. 10 μl of samples was injected onto a ZIC-pHILIC column (150 mm×4.6 mm; 3.5 μm, Merck, Germany) with mobile phase A being 20 mM ammonium carbonate in HPLC grade water (pH 9.2) and mobile phase B being HPLC grade acetonitrile. The eluting gradient was initiated with 80% of B and decreased to 20% B over 30 min. B was further decreased to 8% at 31 min and held at this to 36 min for washing the column. B was then increased to 80% from 37 min and held at this to 46 min for re-equilibration of the column. The flow rate was 0.3 ml/min for the whole eluting gradient. The LC-MS run involved initially two solvent blanks for equilibrating the column, then the four standard metabolite solutions, and then the experimental samples randomly sequenced. The mass spectrometer settings were as follows. The Electospray Ionisation (ESI) interface was operated in a positive/negative polarity switching mode. The spray voltage was 4.5 kV for positive mode and 4.0 kV for negative mode. The temperature of the ion transfer capillary was 275°C and sheath and auxiliary gas flow was 50 and 17 arbitrary units, respectively. The full scan range was 75 to 1200 m/z for both positive and negative modes with settings of AGC target and resolution as Balanced and High (1e6 and 50,000), respectively. The data were recorded using the Xcalibur 2.1.0 software package (Thermo Fisher Scientific). Mass calibration was performed for both ESI polarities before the analysis using the standard Thermo Calmix solution with addition of some additional compounds to cover the low mass range and the signals of 83.0604 m/z (2xACN+H) and 91.0037 m/z (2 x formate-H) were selected as lock masses for positive and negative mode, respectively, during each analytical run.

### Data processing

The LC-MS raw data of the metabolite standard solutions were processed using ToxID 2.1 (Thermo Fisher Scientific Inc., Hemel Hempstead, UK) with ± 3 ppm (parts per million) mass accuracy tolerance with both ESI positive and negative modes. The generated extracted ion chromatograms of metabolite standards on different columns were visually evaluated with respect to peak shapes and their retention times are detailed in S1 Table.

The data processing for biological samples initially involved centroiding and converting vendor-specific raw LC-MS files into the mzXML open format. Chromatographic peaks in these files were extracted using the detection algorithm from XCMS [17] and stored in corresponding PeakML files [18]. Subsequently, PeakML files representing replicates were aligned and combined using mzMatch.R [19] after filtering out all peaks that were not reproducibly detected. The combined PeakML files were subjected to additional noise filtering and gap-filling for peak quality control. Finally the generated CSV files were imported into IDEOM for metabolite putative identification based on accurate mass (±3 ppm) and retention time prediction [20]. The lipids and peptides were excluded from the lists of putatively identified metabolites in the biological samples.

### Metabolite identification

In order to confirm identity of metabolites to the metabolic standards initiative (MSI) level 1 [21], the two orthogonal methods of retention time matching (to ± 0.2 min) against a standard and accurate mass to < 3 ppm were used. Metabolites indentified to level 2 were identified by accurate mass to < 3 ppm and also by predicted retention time [22]. A subset of metabolites identified to level 2 which proved to be biologically interesting to this study but for which no authentic standards were available were identified to level 1 by obtaining interpretable MS^2^ spectra. MS^2^ was carried out by using a Surveyor HPLC system combined with a LTQ-Orbitrap mass spectrometer from Thermo Fisher Scientific (Bremen, Germany) by using Collision Induced Dissociation (CID) at 35 V with the activation Q 0.25 and activation time 30 ms. The same LC gradient programs described above were used and the selective MS^2^ fragments were accurate to ± 5 ppm. The obtained MS^2^ data were compared with the standard spectra in the METLIN database or interpreted if there were no published standard spectra available.

### Normalisation of metabolite levels

The levels of the metabolites in the biological samples are expressed as intensity/2 x 10^6^ promastigotes (parasite extracts) or intensity/2 μl medium (media samples). To ensure validity of comparisons between samples, the protein content of the parasite pellets collected were determined according to the Bradford procedure (BioRAD), using bovine serum albumin as the protein standard.

### Statistical analysis

Principal Component Analysis (PCA) was carried out by using SIMCA-P 13 (Umetrics, Sweden). Prior to PCA, the data were mean-centered and unit variance (UV) scaled. The Student’s T test was performed for all individual putatively identified metabolites: a *p* value smaller than 0.05 (p<0.05) was considered significant; GraphPad Prism 4 was used for plotting the graphs.

### Quantification of confirmed metabolites of interest

In order to determine the concentrations of amino acids in the spent media, a mixture of amino acids (Sigma Aldrich, Product No. A9906) was diluted with the cell extraction buffer chloroform/methanol/water (20/60/20, v/v/v) to give final concentrations at 50, 5, 0.5, 0.05 and 0.005 μmol/L for each compound. For other metabolites of interest (including phenyl pyruvate, phenyl lactate, 4-hydroxyphenyl pyruvate, 4-methyl-2-oxopentanoic acid, 3-methyl-2-oxopentanoic acid, hydroxyisovaleric acid and 3-(4-hydroxyphenyl) lactate), calibration series were prepared with concentrations at 10000, 1000, 100, 10, 1 ng/ml in chloroform/methanol/water (20/60/20, v/v/v). To take into account medium matrix effects, the calibration series for tryptophan was prepared with *L*. *major* spent medium (which lacked detectable tryptophan) and indole-3-pyruvate, indole-3-lactate and indole-3-acetate with complete medium; 75 μl of the diluted samples were then extracted with 300 μl of the medium extraction buffer—chloroform/methanol (20/60, v/v). Due to the poor chromatographic performace in HILIC mode, some of the metabolites (phenyl lactate, 4-methyl-2-oxopentanoic acid, 3-methyl-2-oxopentanoic acid, hydroxyisovaleric acid and 3-(4-hydroxyphenyl) lactate) were quantified using a reversed phase LC method which was developed in our previous study [23], although the initial observation of differences was based on the data from chromatography on ZICpHILIC.

### Analysis of enzymes involved in tryptophan metabolism

Promastigotes from day 3 and day 6 cultures were harvested, washed in ice-cold PBS, and resuspended in lysis buffer (25 mM sucrose, 0.25% Triton X-100, 10 μM E-64, 1 mM phenanthroline, 20 mM pepstatin A, 0.2 M PMSF) at 5 x 10^8^/ml (day 3 cultures) and 10^9^/ ml (day 6 cultures). They were lysed by aspirating 4 times with a Gilson pipette and the extracts were cleared by centrifugation at 14,000 x g for 20 minutes at 4°C and stored at -80^°^C. The protein concentration was determined using the Bradford assay (Bio-Rad).

Tryptophan aminotransferase activity was determined by the method of Szkop et al. [24]. 0.5 ml of 200 mM Tris HCl, pH 7.5, containing 10 mM tryptophan, 5 mM oxaloacetate and 0.04 mM pyridoxal-5-phosphate was incubated for 3 min before adding 50 μl of promastigote extract. 100 μl samples were taken after a 2, 5, 10 and 20 min incubation and mixed with 200 μl of Salkowski’s reagent (10 mM FeCl_3_ in 30% (v/v) H_2_SO_4_). 200μl aliquots were then transferred to a microtitre plate and incubated in the dark for 10–30 min at room temperature. The absorbance at 530 nm was measured in a SpectroMax plate reader and the indole-3-pyruvic acid concentration was determined using a calibration curve that was linear for concentrations of the authentic standard (Sigma) ranging from 2 to 100 μM. The reaction was dependent on tryptophan, oxaloacetate and the promastigote extract.

Malate dehydrogenase (MDH) activity was determined in a 1 ml reaction containing 200 mM Tris HCl, pH 7.5, 5 mM oxaloacetic acid, and 200μM NADH. Reactions were started by addition of 50 μl promastigote extract containing 20–60 μg protein and the rate of reaction determined by monitoring the absorbance at 340 nm. The specific activity in nmol/min/mg protein was calculated using the extinction coefficient for NADH of 6.22 mM^-1^ cm^-1^. Indole-3-lactate dehydrogenase (Ind-LDH), phenyl-lactate dehydrogenase (Phe-LDH) and lactate dehydrogenase (LDH) activities were determined by substituting 5 mM oxaloacetate with 1 mM indole-3-pyruvate, 1 mM phenyl pyruvate or 1 mM sodium pyruvate, respectively. Indole-3-lactate dehydrogenase activity was also assayed in the forward reaction using 5 mM indole-3-lactate and 0.2 mM NAD^+^. Reactions were carried out in 1 ml Tris HCl, pH 7.5, and the rate was determined by monitoring absorbance at 340 nm.

### Measurement of nitric oxide and ammonia in media samples

Media samples from day 6 cultures of the different *Leishmania* species (3 biological replicates) were obtained by taking the supernatants after centrifugation of cultures at 1800 x g for 10 min at 4°C, passing them through a 200μm filter and deproteinating them using a 3000 molecular mass cut off spin column (Vivaspin 500). Nitric oxide (˙NO) production was measured by quantifying ˙NO metabolites in 50 μl samples in a 200 μl reaction using a nitrate/nitrite assay kit (Sigma) with a standard curve of 25–100 μM nitrite. Ammonia concentrations in 50 μl medium samples were measured in 1 ml reactions using an ammonia assay kit (Sigma), with a standard curve ranging from 0.2–15μg/ml ammonia.

### Growth of *Leishmania* in medium containing different concentrations of essential amino acids

HOMEM without amino acids was produced (by Invitrogen) according to the formula in Table A in S3 Table. To prepare medium deficient only in ‘essential’ amino acids, L-glutamate (Invitrogen) and MEM non-essential amino acids (Invitrogen) were added to this to provide the amino acid composition shown in Table B in S3 Table. This modified HOMEM (designated mHOMEM) lacked the 12 ‘essential’ amino acids Phe, Trp, Tyr, Leu, Iso, Val, His, Arg, Lys, Thr, Met and cystine. Medium lacking just tryptophan or arginine were prepared by addition of appropriate amino acid solutions to mHOMEM. Amino acids were removed from FCS by dialysis overnight two times against a 10x volume of PBS at 4^°^C using a 3.5 kDa cut-off membrane, yielding dialysed FCS (dFCS). Promastigotes from day 6 cultures in HOMEM with 10% (v/v) FCS were washed twice by centrifugation at 1800 x g for 10 min at 4°C and resuspension to the initial volume in PBS. Cultures were initiated with the washed promastigotes at 2.5 x 10^5^ cells/ml in the various media. Growth was monitored by determining cell densities at intervals using a haemocytometer.

## Results

### Comparison of the metabolomes of three *Leishmania* species and their spent media

We have used a ZIC-HILIC column in an analysis of extracts from *L*. *donovani* previously [16], whereas two HILIC methods of analysis (ZIC-HILIC and ZIC-pHILIC columns) were compared for their usefulness in the analysis of metabolites in *L*. *major* [25]. The aim was to achieve the widest possible coverage of metabolites using a single column in order to maximise efficiency of detection procedures and thus be able to apply them to a large number of samples. Polar compounds and lipids were detected, however accurate identification of lipids could not be achieved without extensive MS^2^ experiments because of the large number of isomers possible for a given molecular weight. We therefore decided to focus on polar compounds and lipids were excluded from the analysis. This analysis showed clearly that for analysis of the metabolite differences between *Leishmania* species involving a single chromatographic method, the ZIC-pHILIC method is preferable to that with the ZIC-HILIC column, although this column remains valuable for compounds such as polyamines. Thus ZIC-pHILIC method was chosen for the general profiling of the *Leishmania* species extracts.

**Fig 1:**
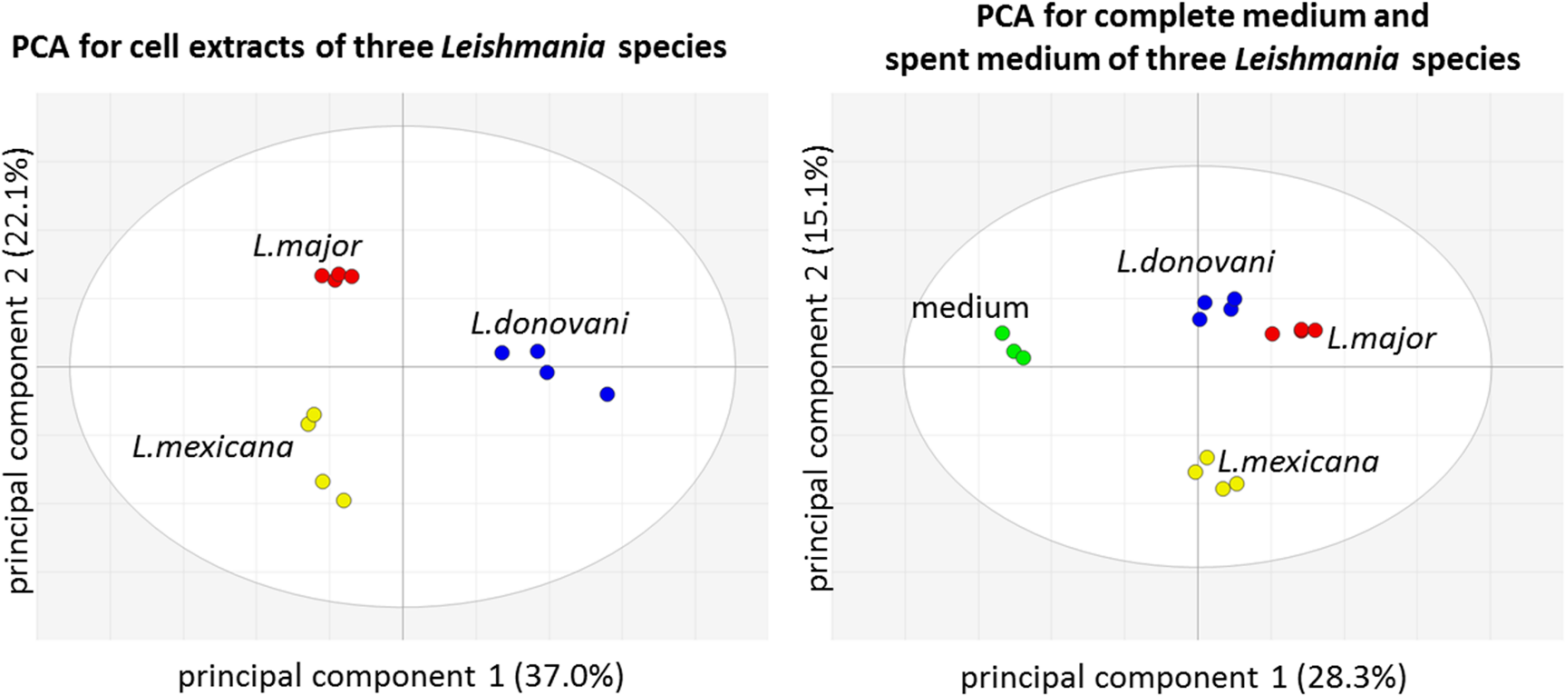
Principal Component Analysis of media and parasite samples. Principal Component Analysis (PCA) for cell extracts of three *Leishmania* species (left, first two components explain 59.1% of the total variance) and for complete medium and spent media of three *Leishmania* species (right, first two components explain 43.4% of the total variance). Key: blue, *L*. *donovani*; red, *L*. *major*; *y*ellow, *L*. *mexicana*; green, complete medium. The confidence ellipses are based on Hotelling’s T^2^ = 95% for both score scatter plots.

The metabolomes putatively identified by applying the ZIC-pHILIC analytical methodology to extracts of promastigotes of three *Leishmania* species and their spent media were distinctly different overall as shown by the PCA plots of the data (Fig 1). The data obtained are fully detailed in S4 and S5 Tables; these include all putatively identified metabolites, identified by accurate mass to < 3 ppm and by predicted retention time, as well as metabolites confirmed to MSI level 1 by matching their retention times (± 0.2 min) against standard compounds or by MS^2^ fragmentation in addition to accurate mass (the full list of confirmed metabolites is given in S2 Table). Spectra of compounds characterised to level 1 by using MS^2^ were either compared to spectra in the Metlin database or spectra were interpreted. S4 and S5 Tables detail the fragments obtained by MS^2^ for metabolites of interest and S1 Fig shows the fragmentation pathway for arginic acid as an example of MS^2^ spectrum interpretation. Using these approaches, we confirmed the identity of 153 metabolites in the *Leishmania* extracts and 137 metabolites in the spent media to MSI level 1, whereas there remained 459 and 548 metabolites identified putatively in the respective samples on the basis of accurate mass and retention time predicted by IDEOM (Table 1). The metabolites confirmed and putatively detected comprise a diverse range of structures including purines, pyrimidines, amino acids and their metabolites, sugars, sugar phosphates and intermediates in glycolysis and organic acids.

**Table 1:** Numbers of metabolites confirmed and putatively identified in cell extracts and spent media.

	Cell extracts		Spent media	
	Confirmed(std, MS/MS)	Putative	Confirmed(std, MS/MS)	Putative
Metabolites after filteringlipids and peptides	153 (135, 18)	459	137 (119, 18)	548
2-fold difference frominitial medium	na	na	79 (63, 16)	173
No change from initialmedium	na	na	58 (56, 2)	375
No clear speciesspecificity	48 (43, 5)	222	21 (18, 3)	53
Clear species specificity (>2-fold change)	105 (92, 13)	237	58 (45, 13)	120
Clear species specificity(>3-fold change)	64 (55, 9)	161	43 (32, 11)	87
Clear species specificity(> 4-fold change)	52 (45, 7)	129	39 (30, 9)	68

**Key:** na, not applicable; std, using standards; the data on species specificity for the spent media are for the metabolites (79 confirmed, 173 putative) showing at least 2-fold differences from the initial medium.

The most abundant intracellular and extracellular metabolites, based on relative MS response, are shown as heatmaps that clearly indicate similarities and also differences between the three species (S2 and S3 Figs) and the full list of metabolites, confirmed and putatively identified, which are significantly increased or depleted in the spent growth media compared with the original complete medium are detailed in S5 Table.

As the main focus of this analysis was to discover differences between the metabolomes of the three species that could provide insights into the biological characteristics of the species themselves and the diseases that they cause, we concentrated on those metabolites for which clear differences were found in intensity between equivalent samples from the three species. In this study we have arbitrarily taken a 2-fold difference as the criterion for difference, although we focused more on those metabolites showing a bigger variation. Of the metabolites confirmed in identity and putatively identified, 105 and 237, respectively, were different between the extracts of at least two species, whereas 58 and 120, again respectively, differed in their abundance in the spent media (see Table 1); the full datasets are presented as S4 (*Leishmania* extracts) and S5 (spent media) Tables.

Our interpretative analysis has concentrated mainly upon those confirmed metabolites that differed in abundance at least 3-fold between the extracts of different species and also those that were consumed from the culture media or released into it in large amounts and there were species differences of at least 3-fold in the consumption/release. These metabolites are listed in Tables 2 and 3. The concentrations of some metabolites differed quite considerably between species, and the trend of variation was not towards higher concentrations in any particular species which gives reassurance that the data do not simply reflect differences in cell volume between species. We addressed this issue by normalising the intensities against the protein content of the extracts, on the rationale that protein content is a good measure of cell volume (as done previously [10]). Cell extracts of *L*. *donovani*, *L*. *major* and *L*. *mexicana* were found to have similar protein contents of 124 ± 17, 99 ± 25 and 85 ± 5 μg per 4 x 10^7^ cells, respectively, indicating that their cell volumes differed by less than 35%. Thus assigning significance to metabolites differing by more than 2-fold between species more than allows for any possible influence of differences between cell volumes. Comparing intensity data normalised to cell protein content showed that all of those in Table 2 still differ by 2-fold and 87% still differ by 3-fold, thus confirming the robustness of the findings. Some metabolites that differ by <3-fold based on cell volume probably differ by >3-fold if the data are normalised to cell protein, but we have not added them to Table 2 to avoid undue complexity of the datasets. The metabolites that differ are from a diverse range of metabolic pathways and the data strongly support the contention that different *Leishmania* species are quite distinct in their metabolism. The data for metabolites in the spent media also are in agreement with this idea. They confirm the utilisation by the parasites of some purines and pyrimidines, but again show some species specificity. Interestingly, the use of sucrose is much less by *L*. *mexicana* than the other two species. The data also show large variations in metabolite release into the culture medium most notably of succinate and deoxyribose. Glycerol and nicotinate are consumed by *L*. *major* but apparently released into the growth medium by the other two species. The variation between species is not consistent with simply a difference in growth rate or rate of differentiation to metacyclic promastigotes as the selective consumption/release of particular metabolites differed between species. Moreover, the data on growth rates showed those of individual species were relatively similar to each other indicating that the cultures were in the same growth phase on day 6 when the extracts were prepared (S4 Fig). It is not possible to monitor the relative rates of metacyclogenesis of the three species as there is no accepted method for distinguishing the different forms of promastigotes that occur in cultures. However, to provide some information on this we did compare the metabolomes in promastigotes that had been cultured for only 3 days and showed that the main differences detected between species in the day 6 analyses detailed in this paper were also present in the day 3 samples (data not shown).

**Table 2:** Metabolites of confirmed to MSI level 1 in promastigote extracts that differed by at least 3-fold in intensity between two species.

Metabolite	Mass	Ldon/Lmaj	Ldon/Lmex	Lmaj/Lmex
Glycine	75.0321	2.73	4.25	1.56
Serine	105.0426	1.69	4.44	2.63
Glycerate	106.0266	22.11	16.78	0.76
Imidazole-4-acetaldehyde	110.0480	4.93	2.11	0.43
Cytosine	111.0433	12.66	6.63	0.52
Proline	115.0634	0.22	0.12	0.57
Threonine	119.0583	8.49	2.13	0.25
Nicotinate	123.0320	0.79	0.11	0.14
Imidazole-4-acetate	126.0428	1.11	0.12	0.11
5-Oxoproline	129.0428	1.43	7.47	5.23
Pipecolate	129.0789	1.28	0.09	0.07
3-Methy2-oxopentanoic acid/4-Methy2-oxopentanoic acid	130.0631	0.59	2.90	4.90
trans-4-Hydroxy-proline	131.0582	2.63	3.32	1.26
Asparagine	132.0534	2.35	3.05	1.30
*Deoxyribose	134.0580	3.15	7.37	2.34
Homocysteine	135.0353	1.61	5.68	3.53
Hypoxanthine	136.0385	5.15	0.37	0.07
Ethanolamine phosphate	141.0192	1.87	0.23	0.12
2-Oxoglutarate	146.0216	1.95	5.97	3.07
O-Acetyserine	147.0532	2.28	3.17	1.39
*Ribose	150.0529	2.67	4.90	1.84
Xanthine	152.0335	16.91	0.21	0.01
*Xylitol	152.0686	4.76	3.73	0.78
Orotate	156.0172	0.29	0.12	0.43
Imidazole lactic acid	156.0535	4.72	2.06	0.44
proline carbamate	158.0690	9.18	2.62	0.29
N-acetyl valine	159.0894	18.61	39.95	2.15
N-gamma-Acetyldiaminobutyrate	160.0847	9.35	3.98	0.43
O-Acetyhomoserine	161.0689	16.70	1.52	0.09
Carnitine	161.1051	7.89	1.50	0.19
D3-phenyllactate	166.0631	3.42	8.28	2.42
3-(3-Hydroxy-phenyl)-propanoic acid	166.0632	4.14	11.59	2.80
N-Acetyleucine	173.1053	8.76	5.47	0.62
Citrulline	175.0957	5.18	2.45	0.47
*Sorbitol	182.0793	8.26	3.05	0.37
N-Acetylglutamine	188.0795	1.66	4.92	2.97
Gulonate	196.0585	2.41	30.95	12.83
Indole-3-lactate	205.0741	4.01	2.93	0.73
Deoxyuridine	228.0748	INF	INF	0.00
Ribose 5-phosphate	230.0192	0.75	4.92	6.57
Cystine	240.0240	4.06	1.31	0.32
Uridine	244.0696	2.11	3.47	1.64
p-aminobenzoyl glutamate	266.0904	INF	15.50	0.00
N-(Arginino)succinate	290.1228	16.71	0.39	0.02
Glutathione	307.0841	3.62	1.03	0.28
Ribose 1,5-bisphosphate	309.9858	0.12	4.27	36.62
GMP	363.0586	4.15	1.93	0.47

**Key**: Ldon, intensity in *L*. *donovani* extract; Lmaj, intensity in *L*. *major* extract; Lmex, intensity in *L*. *mexicana* extract.

* Matches retention time of standard but ability of the method to separate all the isomeric possibilities not established.

**Table 3:** Metabolites confirmed to MSI level 1 in spent media samples that differed by at least 3-fold in intensity between two species.

Metabolite	Mass	Ldon/Lmaj	Ldon/Lmex	Lmaj/Lmex	M/L.don	M/L.maj	M/L.mex
Glycerol	92.04724	17.31	1.88	0.11	0.22	3.75	0.41
Glycerate	106.0266	3.24	5.04	1.56	0.15	0.48	0.76
Cytosine	111.0433	10.08	5.81	0.58	0.71	7.14	4.12
Proline	115.0634	0.57	0.07	0.12	7.57	4.30	0.51
3-Methy2-oxobutanoic acid	116.0474	0.09	0.61	6.47	0.18	0.02	0.11
Succinate	118.0267	1.24	4.55	3.67	0.01	0.01	0.03
Nicotinate	123.032	2.73	0.07	0.03	0.58	1.59	0.04
*3-Methy2-oxopentanoic acid/4-Methy2-oxopentanoic acid	130.0631	0.83	6.69	8.09	0.02	0.02	0.16
Aspartate	133.0376	5.01	1.38	0.28	2.37	11.86	3.27
Deoxyribose	134.058	0.44	4.20	9.63	0.01	0.00	0.03
Hypoxanthine	136.0385	8.60	14.32	1.67	83.28	716.28	1192.67
4-Hydroxyphenylethanol	138.0681	0.03	0.53	16.57	0.78	0.03	0.42
Glutamate	147.0533	23.34	2.01	0.09	2.02	47.25	4.07
[FA oxo,methyl(4:0)] 2-oxo-4-methylthio-butanoic acid	148.0195	0.55	2.86	5.18	0.00	0.00	0.00
Xanthine	152.0335	34.08	1.00	0.03	20.02	682.19	20.03
N-acetyl valine	159.0894	3.18	2.03	0.64	0.16	0.51	0.33
Phenylpyruvate	164.0475	5.24	1.76	0.34	0.00	0.01	0.00
3-phenyllactate	166.0631	1.23	5.18	4.21	0.00	0.00	0.00
3-(3-Hydroxy-phenyl)-propanoic acid	166.0632	1.31	4.70	3.58	0.00	0.00	0.00
5-Guanidino-2-oxopentanoate	173.08	0.03	0.06	2.21	1.47	0.04	0.09
N-Acetylornithine	174.1004	5.62	1.50	0.27	1.74	9.81	2.61
Indole-3-acetate	175.0634	0.04	1.16	32.34	0.66	0.02	0.77
Arginic Acid	175.0957	0.11	0.24	2.22	0.00	0.00	0.00
Hippurate	179.0583	0.00	0.00	0.00	INF	INF	INF
3-(4-Hydroxyphenyl)pyruvate	180.0425	3.47	1.70	0.49	0.00	0.01	0.00
2-Phospho-glycerate	185.993	0.24	1.30	5.47	0.00	0.00	0.00
Tryptophan	204.0897	1016.88	1.40	0.00	1.99	2024.85	2.78
Cystathionine	222.0675	0.78	2.88	3.69	0.02	0.01	0.05
Deoxyuridine	228.0748	21.20	28.21	1.33	1.53	32.45	43.19
Cytidine	243.0854	5.95	12.44	2.09	1.47	8.74	18.28
Uridine	244.0696	INF	INF	0.00	22.63	INF	INF
Adenosine	267.0964	7.31	1.49	0.20	0.43	3.11	0.64
Guanosine	283.0917	0.00	0.00	0.00	INF	INF	INF
Sucrose	342.1162	0.00	0.00	0.00	INF	INF	6.82
AMP	347.0635	0.00	0.00	0.00	INF	INF	INF
S-Adenosyhomocysteine	384.1214	0.28	2.02	7.28	0.00	0.00	0.00
Raffinose	504.1696	3.33	0.76	0.23	1.66	5.51	1.27

**Key:** INF, metabolite absent from spent medium but present in original medium; M, intensity in complete medium; Ldon, intensity in *L*. *donovani* spent medium; Lmaj, intensity in *L*. *major* spent medium; Lmex, intensity in *L*. *mexicana* spent medium; the ratios of the species is based on the ratio for each with the complete medium.

*Combined acids co-eluting on ZIC-pHILIC. Separately quantified using C18 AR column.

**Fig 2:**
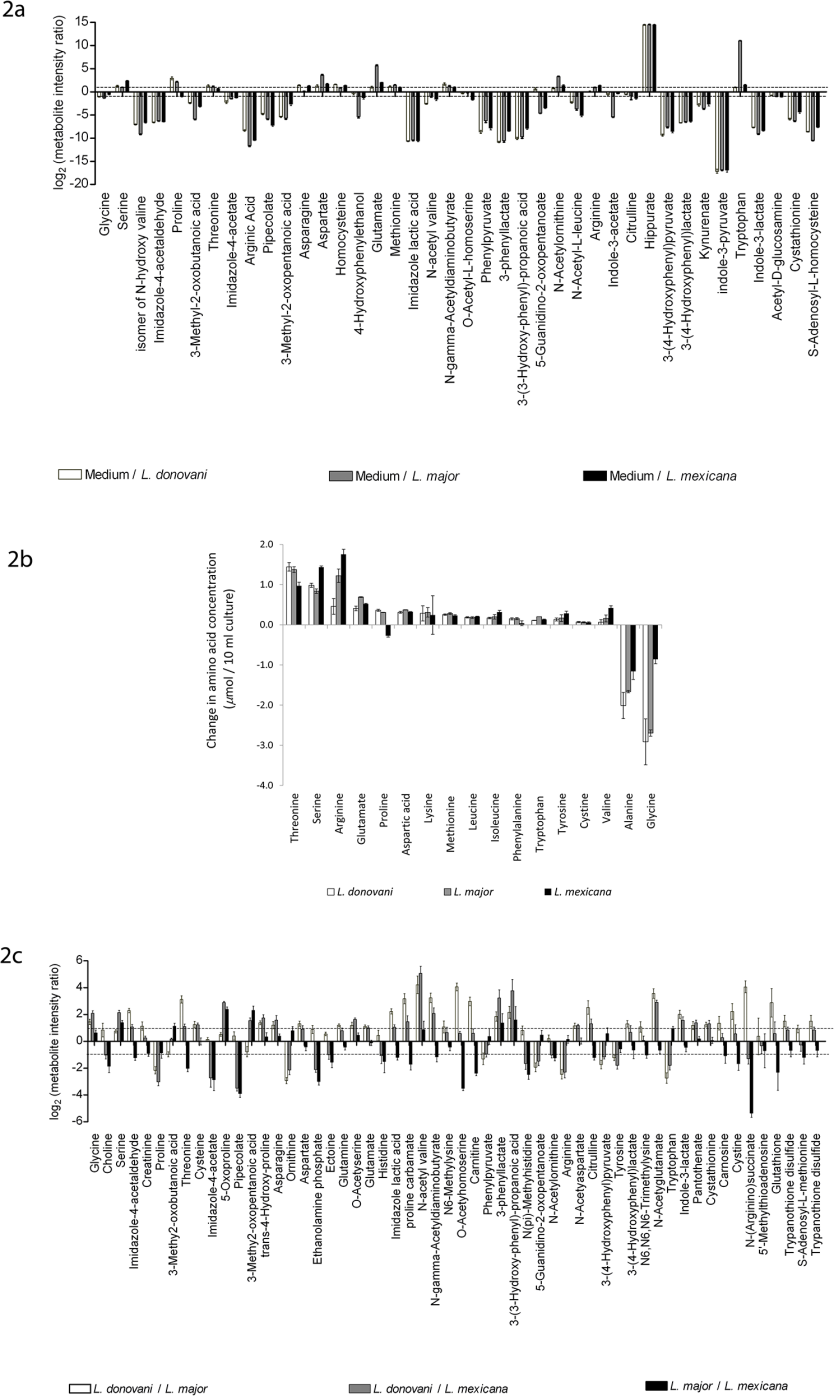
Comparative consumption of amino acids from media and their intensities in parasite samples. Ratios of amino acids and some derivatives in spent media in comparison with complete medium (A) and between extracts of three *Leishmania* species (C). The ratios of amino acid intensity for complete medium (Medium) to spent medium from each *Leishmania* are presented as log_2_. Only amino acids and their derivatives which differ from the medium by > log_2_1 for at least one species (2A) or between species by > log_2_1 (2C) are included. Key: black bar, Medium/*L*.*mexicana*; white bar, Medium/*L*. *donovani*; grey bar, Medium/*L*. *major*. For extracts, intensity ratios between each pair of *Leishmania* species are expressed as log_2._ Key: white bar, *L*.*major*/*Lmexicana;* black bars, *L*. *major*/*L*. *donovani*; grey bars, *L*.*mexicani*/*L*.*donovani*. The amounts of amino acids consumed from the medium are also detailed for the three species (B).

Many of the metabolites differing both intracellularly and extracellularly are amino acids or derivatives of them. The identities of all amino acids in the biological samples were confirmed to MSI level 1, which allowed for a full comparison of these compounds. The concentrations of amino acids themselves in the unused medium are relatively high, thus supplying a rich source for the parasites to consume. Comparing the observed intensities measured for each in the original medium and the spent media after parasite growth showed that, for most, the amount consumed was less than half of the original amount available (log_2_ratio <1 in Fig 2A, hence many amino acids are not shown in the figure). However, for a few this was not the case and also there was some clear species-specificity (Fig 2A). Notably, far greater percentages of the aspartate, glutamate and tryptophan in the medium were used by *L*. *major* than by either of the other two species, the tryptophan being totally consumed. In contrast, proline was apparently used by *L*. *major* and *L*. *donovani* but released by *L*. *mexicana*, whereas each of arginine and serine were more greatly consumed by *L*. *mexicana* than by the other species (Fig 2B). Some amino acid derivatives were also released into the medium, including arginic acid, pipecolate, phenylpyruvate, phenyllactate, 4-hydroxyphenyl pyruvate, 3-(4-hydroxyphenyl) lactate, indole-3-pyruvate, indole-3-lactate and cystathionine (Fig 2 and Table 3). To gain a better insight into the quantitative use of the amino acids, we generated standard curves for each amino acid (S5 and S6 Figs) and used these to estimate the levels of each in the spent media (Fig 2B and S6 Table). These data reiterated the species differences in the use of some amino acids as described above, but also showed that in quantitative terms other amino acids were also used and released in large amounts. L-alanine was released in quantity by all three species and glycine was also released in quantity by *L*. *donovani* and *L*. *major*. Most other amino acids were consumed, but the molar amounts used covered a wide range (for instance, 140 μM L-threonine was consumed by *L*. *donovani* whereas only 5 μM L-valine was used by the same species) and there were quite large differences between species although all species consumed higher levels of serine, threonine and arginine than other amino acids. The clear differences in utilisation of some amino acids by the three species was not reflected in the levels of the compounds in the extracts from the cells (Fig 2C).

### Uptake and catabolism of tryptophan

**Fig 3:**
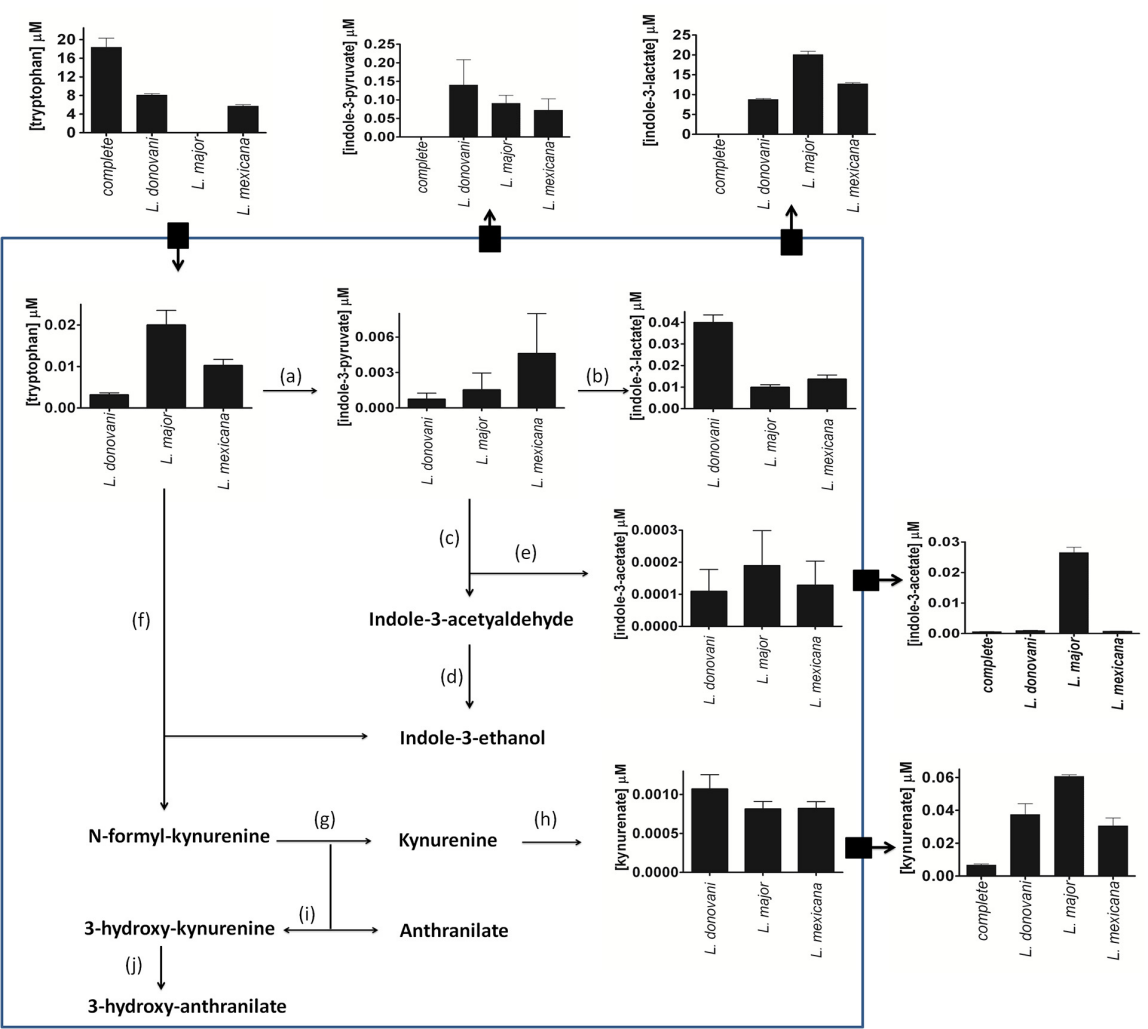
Tryptophan catabolism in the three *Leishmania* species. Key: **a**, tryptophan aminotransferase or L-amino oxidase [48]; **b**, indole-3-lactate dehydrogenese [48]; **c**, phenyl-pyruvate decarboxylase or indole-3-pyruvate decarboxylase (LmjF34.3250); **d**, indole-3-acetylaldehyde reductase; **e**, indole-3-acetylaldehyde oxidase (LmjF26.2280); **f**, indole 2,3 deoxygenase or tryptophan 2,3 deoxygenase; **g**, acrylformamidase; **h**, kynurenine oxoglutarate transaminase or cysteine-S-conjugate *β*-lyase or glutamine phenylpyruvate transaminase (LbrM33_V2.1600); **i,** kynurenine-3-monoxogenase; **j**, carboxy lyase.

Tryptophan was shown to be totally consumed by *L*. *major* but less so by the other species, thus we carried out a more detailed quantitative analysis of tryptophan and its catabolic products. We looked for likely products of tryptophan catabolism, both in the cell extracts and also in the spent media, and confirmed the identity of metabolites found by comparison with authentic standards. We then quantified the amounts of the metabolites using standards to produce calibration curves (S6 Fig). The data generated are presented in Table 4 and schematically in Fig 3. This analysis showed that little tryptophan was catabolised via the kynurenate pathway in any of the three species and that most was transformed to indole-3-lactate and released into the medium. Indole-3-lactate was not detected in the uninfected control media samples confirming that it was synthesised in *Leishmania* and not the result of chemical conversion.

**Table 4:** Tryptophan metabolites recovered in the spent media.

	Complete medium	*L*. *donovani* spent medium		*L*. *major* spent medium		*L*. *mexicana* spent medium	
	mean ±SD	mean ± SD	%	mean ±SD	%	mean ±SD	%
Indole-3acetate	0.00 ±0.00	0.00 ±0.00	0.0	0.17 ± 0.01	0.9	0.00 ± 0.00	0.0
Indole-3-lactate	0.04 ±0.01	8.79 ±0.45	47.9	20.05 ± 1.68	109.3	12.75 ± 0.52	69.5
Indole-3-pyruvate	0.00 ±0.00	0.14 ±0.14	0.8	0.09 ± 0.04	0.5	0.07 ± 0.06	0.4
Kynurenate	0.01 ±0.00	0.04 ±0.01	0.2	0.06 ± 0.00	0.3	0.03 ± 0.01	0.2
Tryptophan	18.33 ±3.87	8.08 ±0.61	44.1	0.02 ± 0.00	0.1	5.70 ± 0.62	31.1

**Key:** %, % tryptophan in complete medium recovered in the spent medium as this metabolite; concentrations in μM.

### Analysis of the tryptophan degradation pathway in *L*. *major*

We hypothesised that the extensive consumption of tryptophan and almost equimolar generation of indole-3-lactate by *L*. *major* was mediated by initial transamination of tryptophan to indole-3-pyruvate followed by reduction of this to indole-3-lactate and that these were mediated by the activities of an aminotransferase and hydroxyacid dehydrogenase, respectively (as indicated in Fig 3). To test this, we assayed for tryptophan aminotransferase activity in protein extracts from *L*. *donovani*, *L*. *major* and *L*. *mexicana* promastigotes after 3 and 6 days of *in vitro* growth, using tryptophan (10 mM) and α-ketoglutarate, oxaloacetate, or pyruvate (each at 5 mM) as co-substrates. The reaction was monitored by determining the generation of indole-3-pyruvate [24]. *L*. *donovani* extracts at day 3 had the highest activity, with oxaloacetate being the best co-substrate, with much lower activity being detectable in extracts of *L*. *major* (Table 5). In contrast, assays using *L*. *major* extracts to test for indole-3-lactate dehydrogenase activity in either the forward or reverse direction detected no activity (level of detection, 25 nmol/min/mg protein) and reactions with phenyl pyruvate (1 mM) or pyruvate (5 mM) and NADH also showed no activity. However, incubation of rabbit muscle lactate dehyrogenase (Roche) with pyruvate or phenyl pyruvate and NADH as a positive control produced a measurable rate (data not shown), although this enzyme also showed no activity with indole-3-pyruvate. As a second positive control, we confirmed that extracts of *Leishmania* had malate dehydrogenase activity converting oxaloacetate to malate with NADH as the reducing agent with specific activities of 49, 49 and 18 nmol/min/mg protein for *L*. *donovani*, *L*. *major* and *L*. *mexicana*, respectively.

**Table 5:** Tryptophan:oxaloacetate aminotransferase activities of *Leishmania* cell extracts.

	Day 3 μmol/min/ mg protein	Day 6 μmol/min/ mg protein
*L*. *donovani*	1.68 ± 0.48	0.49 ± 0.16
*L*. *major*	0.07 ± 0.04	0.22 ± 0.02
*L*. *mexicana*	0.21 ± 0.10	0.09 ± 0.01

Extracts were prepared from *Leishmania* cultures after 3 and 6 days growth at 26°C and assayed for tryptophan aminotransferase activity with oxaloacetate as the co-substrate. Indole-3-pyruvate produced was quantified using the method of Szkop *et al*. [24]. Activities represent the mean of 3 independent reactions ± standard deviation.

### Uptake and catabolism of arginine

**Fig 4:**
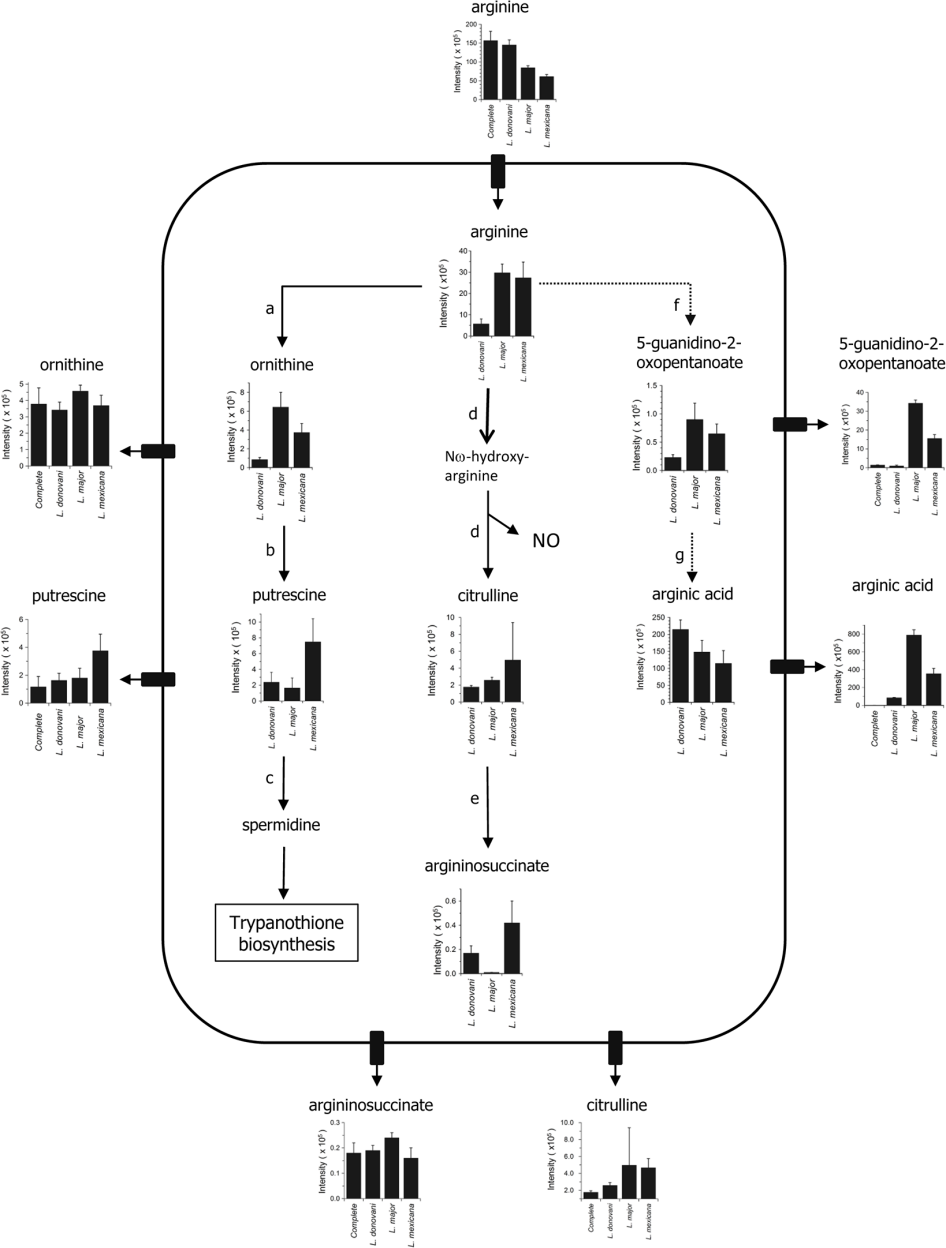
Arginine metabolism the three *Leishmania* species. The histograms give the relative levels of metabolites in cell extracts and supernatant media (data are peak intensity (x 10^5^) per 10 μl sample (equivalent to 2 x 10^5^ cells) or 2 μl of medium). Data are presented in full detail in S7 Table. Key: **a**, arginase (LmjF.35.1480); **b**, ornithine decarboxylase (LmjF.12.0280); **c**, spermidine synthase (LmjF.04.0580); **d**, nitric oxide synthase; **e**, argininosuccinate synthase (LmjF.23.0260); **f**, arginine aminotransferase; **g**, hydroxyacid dehydrogenase. A soluble nitric oxide synthase has been reported for *L*. *donovani* [66] and purified from *L*. *braziliensis*, *L*. *chagasi* [74] and *L*. *amazonenzesis* [74,75]. Genes for arginine aminotransferase and the hydroxyacid dehydrogenase have not been found but these activities are assumed to be present.

Arginine was also used in large amounts and especially by *L*. *mexicana*, which reduced the medium concentration by 175 μM; in contrast *L*. *donovani* only reduced the concentration by 45 μM (S6 Table and Fig 2B). To analyse the fate of this amino acid within the promastigotes, we looked for the presence of potential catabolites both within the cell extracts and also the spent media (Fig 4 and S7 Table). This revealed that a major route of catabolism was to arginic acid, presumably mediated via the catalysis of an aminotransferase and dehydrogenase enzymes in an analogous way to that in which tryptophan is degraded to indole-3-lactate (Fig 3). Interestingly, however, this route appears to have a greater flux in *L*. *major* than in *L*. *mexicana* with more arginine being converted to argininosuccinate and polyamines in the latter parasite.

### Phenylalanine, tyrosine, leucine, isoleucine and valine deamination

Most amino acids in the spent medium were at >50% of their levels in the initial medium (S6 Table). Thus they were not limiting nutrients, but the differing amounts of the various amino acids consumed (Fig 2C) suggested that some must be used in ways in addition to protein synthesis. Analysis of putative metabolites potentially resulting from amino acid degradation suggested that indeed there was significant amino acid degradation (S7 Fig). Further study to confirm identities and quantify amounts showed there to be large amounts of the phenylalanine catabolites, phenylpyruvate and phenyllactate in the spent media from all three species, the total levels of these two metabolites accounting for a significant proportion of the initial concentration of phenylalanine in the complete growth medium and most of that consumed (S8 Table). The levels of the tyrosine metabolites 4-hydroxyphenyl pyruvate and 3-(4-hydroxyphenyl)lactic acid in the spent media were also significant and the same was the case for the acidic metabolites of leucine, isoleucine and valine; 4-methyl-2-oxopentanoic acid, 3-methyl-2-oxopentanoic acid, alpha-hydroxyisovaleric acid, respectively (S8 Table). However, the hydroxy-acid derivatives of the branched chain amino acids were not detected, presumably reflecting the specificity or subcellular location of the hydroxy acid dehydrogenase. Glutamate produced by aminotransferase activity with α-ketoglutarate as the amino acceptor is further catabolised by glutamate dehydrogenase, releasing ammonia. We determined the ammonia concentrations in the media. Complete medium contained 192 μM and the spent media contained amounts in addition to this as follows: *L*. *donovani*, 24 μM; *L*. *major*, 49 μM; *L*. *mexicana*, 85 μM.

### Growth of *Leishmania* promastigotes with different concentrations of amino acids

To determine the requirement of the different *Leishmania* species for an exogenous supply of some amino acids, promastigotes were cultured in media with different amino acid concentrations. Modified HOMEM (mHOMEM) was prepared without the ‘essential’ amino acids Trp, Phe, Tyr, Leu, Iso, Val, Met, Lys, Arg, His, Thr and cystine (Table B in S3 Table) and washed promastigotes inoculated into HOMEM and mHOMEM supplemented with FCS or dialysed FCS (dFCS). FCS is a source of amino acids, peptides and protein; LC/MS analysis showed that dialysis of the FCS with a cut-off of 3.5 kDa removed >95% of the selected amino acids (S8 Fig). Promastigotes of all three species grew equally well in HOMEM with 10% FCS and mHOMEM with 10% FCS even though the concentration of essential amino acids in the mHOMEM medium, derived only from the FCS, was reduced 3 to 5-fold. Replacement of the FCS with dFCS resulted in greatly reduced cell densities being reached and also reduced multiplication rates with all three species, irrespective of whether HOMEM or mHOMEM was used, although the reduction in cell densities achieved was greater with dFCS in all cases. S9 Fig gives the detailed data for *L*. *major*. The cell density of *L*. *major* grown in HOMEM was reduced 2.8-fold (P < 0.01) on day 3 and 10-fold (P < 0.001) on day 6 when 10% FCS was substituted with the dialysed serum (media A and B in S9 Fig). Comparison of growth in HOMEM with 10% dFCS (medium B) and mHOMEM with 10% dFCS (medium D) showed a 2.7-fold (P < 0.001) reduction in titre. These results indicated that growth was limited by loss of growth factors from the dialysed FCS in addition to a small effect from the loss of the essential amino acids. *L*. *major* grown in media with dFCS remained viable as judged by microscopic observation and re-grew when sub-cultured in medium containing FCS (HOMEM or mHOMEM) but not in medium with dFBS.

The spent media samples from days 3 and 6 of the *L*. *major* cultures in HOMEM and mHOMEM with 10% FCS detailed in S9 Fig (media A and C) were analysed by LC/MS and the concentrations of selected amino acids and derivatives were calculated from their mean peak areas with reference to standard curves (S10 and S11 Figs). Aspartate and alanine are examples of ‘non-essential’ amino acids present at the same initial concentrations in both media. As expected, aspartate levels were greatly reduced in day 6 extracts in both media while alanine was released and these trends were also observed in the day 3 extracts (S10 Fig). Also as expected, the concentrations of essential amino acids in medium C, in which they were supplied entirely by the 10% FCS, were 3 to 5-fold lower than in medium A. Despite the differences in availability, however, similar amounts of phenylalanine and arginine were consumed in both cultures A and C; representing a markedly higher percentage of the total initially available in medium C. Similarly, the amount of tyrosine consumed in medium C was some 75% of that in medium A. The situation for tryptophan, however, was different. The use over the first three days was similar in media A and C but by day 6 it had been completely removed from the medium in both cultures A and C; thus the level of tryptophan consumption was 5-fold higher in medium A although growth was similar in both.

To analyse further the requirement of tryptophan for growth, *L*. *major* was grown in media containing 10% or 2% FCS (S12 Fig). The reduction in FCS concentration resulted in only a 2-fold lower final titre in HOMEM-based media (P < 0.001) whilst there was further 2.6-fold reduction in final titre (P < 0.001) when growth was compared in mHOMEM with 2% FCS and HOMEM with 2% FCS; this reduced growth thus can be attributed to the lower concentrations available of essential amino acids. A medium identical to HOMEM except for lacking tryptophan with 2% FCS, which contained only approximately 3 μM tryptophan, supported similar levels of growth as complete HOMEM with 2% FCS, which contained 50 μM tryptophan (S12 Fig). Tryptophan is essential for growth of *L*. *major* [26], but these results show that the amount required (<3 μM) is very much less than the amount consumed (50 μM) when available (S10 Fig).

## Discussion

*L*. *donovani*, *L*. *mexicana* and *L*. *major* cause leishmaniasis with different clinical manifestations, visceral and cutaneous, and associated pathology [27]. Genomic and transcriptomic analyses have not yet provided a full explanation for the distinct species-specific biology of the three species [3,28]. Some distinct biological differences between the three species are, however, known. For instance, the lipophosphoglycan on the surface of the promastigotes is species-specific and is thought to account for differences in their attachment to sand fly midgut and play a role in the transmission to the mammalian host [29,30]. The species-specific gene *A2* is important for visceral infections in mice by *L*. *donovani* [31] and when introduced into *L*. *major* it imparted some of the tissue tropic properties associated with *L*. *donovani* [32]. Nevertheless, the major factors determining the different infection specificities of the three species had not been elucidated. We reasoned that knowledge of the metabolomes of the three species may shed light on key biochemical differences between the species that could be instrumentmental in determining the disease phenotypes in the mammal. Unfortunately it is not currently possible to obtain the disease-causing stage of the parasite, the amastigote, pure and in sufficient numbers to allow comparative metabolomics analyses to be undertaken. Knowledge of the extent to which amastigotes and promastigotes of *Leishmania* differ biochemically is very limited [13,33–36], some differences have been established although the published metabolomics investigations using *L*. *mexicana* amastigotes that were grown *in vitro* suggest that the metabolic differences between the two parasite developmental forms perhaps are relatively few [13,37]. Thus we adopted a pragmatic approach in this study and compared *in vitro* grown promastigotes of the three species in the hope that this would reveal interesting biochemical differences that may be relevant to the amastigote stages too and so provide some insights into the mechanisms underlying the biological specificitiesof the three species. We used equivalent promastigote forms, as far as it is possible to determine with currently available methodologies, to minimise any variations due to growth phase.

As part of the study, we developed the methodology for analysing the metabolomes of the cells. A full description of our optimisation of column types and mobile phase composition for the detection of the maximum number of metabolites is published elsewhere (25). Our method incorporated both reverse phase and HILIC columns and metabolite identity was confirmed by the use of authentic standards and by interpretation of MS^2^ spectra. This enabled us to confirm 165 metabolites to MSI level 1 (S2 Table) that occurred in the cell extracts or spent media. In addition, 604 metabolites were identified putatively on the basis of accurate mass and retention time predicted by IDEOM. Thus the study has provided a foundation methodology that can be applied extensively in future analyses of the metabolomes of pathogens in general.

This study has resulted in extensive information on the metabolites present in both the cells themselves and the media after their growth. Clearly, snapshot data such as those obtained in this study always have to be interpreted carefully and the recent demonstration that *de novo* synthesis of serine, glycine and proline was repressed in the presence of exogenous amino acids [38] provides a good example of the complex interplay that exists between culture conditions and metabolism which may not mirror that which occurs *in vivo*. However, our experimental approach was to compare the three different species in the same growth phase and under the same conditions, with the expectation that differences observed would give a good indication of innate genetic and physiological species-specificities. Indeed, the main purpose and outcome of any global metabolomics analysis is to generate hypotheses that can be tested by detailed biochemical analysis. The current study has revealed many interesting differences between the metabolism of the three *Leishmania* species, particularly in the area of amino acid metabolism. Amino acids are abundant in the gut of the sand fly in which promastigotes multiple naturally, and some are thought to be used as energy sources [39]. They are also thought to be present in large amounts within the parasitophorous vacuole within macrophages in which the parasite resides in the human host [40]. *Leishmania* can synthesize some amino acids *de novo*, but they are auxotrophic for many [41] and require salvaging these from their hosts. Our findings confirm that they are able to do this effectively and, indeed, in quantity for some amino acids (S6 Table). The findings concerning amino acid usage also prompt speculative hypotheses on how the species differences, if they occur also with the amastigotes that are responsible for human diseases, could explain the distinct disease phenotypes resulting from the infections of humans with the parasites.

It is well established that one mechanism whereby some pathogens attenuate the mammalian host’s immune response is through the depletion of amino acids central to immune mechanisms [42,43,44]. Our results which have revealed that promastigotes of the three *Leishmania* species differ extensively in their use of some amino acids (Fig 2A and 2B) suggest that perhaps such a survival mechanism has evolved for *Leishmania*. Moreover, if the species-specificities in amino acid consumption found with promastigotes also occur with amastigotes they could play an important part in determining where each parasite can survive and flourish.

Some of the most interesting findings are the very clear and distinct inter-specific differences in the consumption of tryptophan and arginine, two amino acids that have been shown to be of particular importance in mammalian immune responses [42,43]. *L*. *major* consumed all of the tryptophan from the medium whereas *L*. *donovani* consumed about half and *L*. *mexicana* approximately two thirds (Figs 2 and 3, S6 Table). Tryptophan is an essential amino acid in mammals and, importantly, plays key roles in the immune response, including that against pathogens [42–46]. One anti-pathogen mechanism is for the host to deprive the pathogen of tryptophan, apparently by stimulating the consumption of the amino acid by the host cells via the kynurenine pathway. Such an immune mechanism depends upon the need of the pathogen for tryptophan and its inability to synthesise it. One good example is the case of *Mycobacterium tuberculosis*, and, interestingly, it was recently reported that the pathogen can overcome immunity and so become persistent by activating genes enabling it to synthesise tryptophan *de novo* [47]. *Leishmania* is considered auxotrophic for tryptophan and also lacks the genes for biosynthesis [41,33], although our data indicates that *L*.*major* need relatively low concentrations for survival. Clearly the amino acid is required for protein synthesis, although catabolism to indole-3-lactate had also been reported [48]. Thus the wholescale utilisation of exogenous tryptophan by *L*. *major*, unlike the other two species, raised three immediate questions. Firstly, do amastigotes similarly avidly use this amino acid? If this is the case, then is this wholescale use of tryptophan by *L*. *major* a means of countering the host cell’s immune response? Does the parasite use it in some way beneficial to itself other than simply getting sufficient for its protein needs? The different extent to which the three parasites consumed exogenous tryptophan also suggested that perhaps they metabolised it in different ways and also that they may well have different, at least quantitatively, mechanisms for attenuating the host’s immunity. The latter point is consistent with many immunological studies over many years [46,49–52] and also could help explain why the three species reside in different tissues and thus host cells in mammals.

We investigated whether or not the three species catabolise tryptophan differently and our results show that indole-3-lactate is by far the major end-product in each case and accounts for effectively all of the tryptophan consumed from the medium. Just small amounts of indole-3-acetate, indole-3-pyruvate and kynurenate were released. This pathway to indole-3-lactate is likely to involve aminotransferase and hydroxyacid dehydrogenase enzymes and we detected the former activity in cell extracts of all three species (Table 5). The specific activities, however, did not correlate with the relative rate of consumption of tryptophan by the species, which may therefore be determined either by the rate of uptake of the amino acid or the rate of its conversion to the lactate. We were unable to detect the dehydrogenase activity in cell extracts as previously reported for *L*. *mexicana* [53]. *Leishmania* encodes a cytoplasmic malate dehydrogenase (cMDH) with 50% similarity to the aromatic hydroxyacid dehydrogenase (AHADH) present in *Trypanosoma cruzi* that reduces phenyl pyruvate and 4-hydroxyphenyl pyruvate [53]. However, although the *T*. *cruzi* AHADH is structurally related to cMDH it has no activity with oxaloacetate as a substrate whereas the *L*. *major* cMDH has a high specificity for oxaloacetate indicating that it functions as a malate dehydrogenase [53,54]. The release of aromatic lactates by the three species (Table 4, S8 Table) shows that that an AHADH activity must be present in *Leishmania*, although it may require special conditions for function. AHADH activity catalysing the formation of p-hydroxyphenyl-lactate from 4-hydroxyphenyl pyruvate, the product of tyrosine transamination, was recently detected in *L*. *infantum* cell extracts, although the activity was very low [55]. However the gene for the *Leishmania* AHADH is yet to be identified.

Trypophan degradation via the kyurenine pathway is an important component in the immune response and there have been reports that *L*. *major* modulates this, although with differing conclusions on the importance of the mechanism [33,41,44,]. Indoleamine 2,3 dioxygenase (IDO) seems the key regulatory enzyme of the pathway and inhibition of this is perceived to have potential in anticancer therapies [56,57]. A known inhibitor of IDO is 1-methyl-L-tryptophan (1MLT) and this has been tested for an effect against experimental infections against *Leishmania* [51,52,58], although without yielding consensus data. We looked for an effect of the inhibitor against the parasite itself, with the hypothesis that it may interfere with tryptophan consumption by the parasite even though the parasite apparently lacks a IDO homologue. However, we found that 1MLT has no growth inhibitory activity (IC50 > 1 mM) and also that adding 1MLT to growing cultures at 1 mM did not change metabolism at all as judged by the intracellular and released metabolomes (data not shown).

The outstanding question is why the parasite carries out this catabolism of tryptophan. The amount of tryptophan converted to indole-3-lactate by *L*. *major* promastigotes was more than 10-fold higher than the concentration the parasite required for growth (S10 and S12 Figs). The pathway generates NAD^+^ and so could function as a means of helping to maintain redox homeostasis, although it was reported that addition of exogenous tryptophan had no effect on the intracellular NAD^+^ content [59]. We suggest that the avid utilisation, if also carried out by the intracellular amastigote form, could be a means of depleting the host cell of tryptophan and hence suppressing the T-cell response [51,52] and thus represent a parasite strategy for evading the immune system. Differing use of tryptophan by the three species would be consistent with them having differing matrices of responses for immune evasion and that perhaps they are adapted for survival and growth in different host cells.

Although tryptophan utilisation showed the most extreme species-specificity on a percentage basis, other amino acids were consumed by the three species in very different amounts. A notable example was arginine, with *L*. *mexicana* using 4-fold more than *L*. *donovani*, with *L*. *major* being intermediate. Arginine is considered to be another key metabolite in the mammalian immune response and has been implicated in a number of ways [43,60,61]. One route of catabolism is to generate nitric oxide (˙NO) via nitric oxide synthase (NOS). ˙NO is an important component in the cytotoxic action of macrophages and it has been shown that activated macrophages kill *L*. *major* via ˙NO production [62]. Stimulation of ˙NO production within macrophages has been shown to increase their level of cytotoxicity against canine visceral leishmaniasis [63]. Arginine also affects a mitogen activated kinase (MAP kinase) signalling pathway involved in the innate immune response to microbial pathogens [64,65]. Thus scavenging arginine and so depriving the host cell of this key amino acid could also be a mechanism of immune evasion [60,61] and our data on arginine consumption (S6 Table), if it is reflective of the consumption by amastigotes too, would be consistent with differing in importance of this mechanism between species.

What is the fate of the consumed arginine? We investigated this by analysing possible catabolites. Initial observations suggested that citrulline was secreted in large amounts into the spent medium and was also the most abundant intracellular metabolite in terms of rank order of intensity. This lead us to measure ˙NO production by *Leishmania*, as NOS activity was reported previously [66], but we were unable to detect any ˙NO metabolites (< 25 μM nitrate) in the spent media. On more detailed investigation it became clear that the metabolite thought to be citrulline had a slightly different retention time from the authentic citrulline standard and its MS^2^ spectrum (S1 Fig) was consistent with this metabolite being arginic acid. Citrulline itself was present in only low amounts in the complete medium and spent media (S6 Table). Arginic acid, which is formed via deamination of arginine followed by reduction, is not a substrate for NOS and indeed is considered an end product. We were unable to quantify in molar terms the amount of arginic acid released, but the very high intensity of the peak suggests that it was considerable and this is consistent with the amounts detected of the other arginine catabolites that were quantified (Fig 4). Arginic acid was not found in uninfected media samples and was therefore not produced by chemical degradation. Thus a working hypothesis is that the parasites consume arginine and convert it to arginic acid as a mechanism of reducing the immune response of the host cell. Moreover, the relative intensities of the arginic acid released by the promastigotes of the three species, if also true for amastigotes, would be consistent with this mechanism being more important for *L*. *major* than for the other two species.

Glutamate was also consumed in large amounts, especially by *L*. *major* which almost depleted the medium of all of the amino acid (S6 Table). Recent data for *L*. *mexicana* suggest that glutamate incorporation is minimal under standard *in vitro* culture conditions; this study showed that C^13^-U-glutamate was incorporated into the secreted metabolites CO_2_, succinate, alanine, acetyl-CoA and glutamine, with highest levels released by logarithmic phase promastigotes [13]. Our data show that proline also was released into the medium in quite large amounts by *L*. *mexicana* whereas the other two species consumed large amounts of proline from the medium (S6 Table). It has been reported that proline is used as an energy substrate by *L*. *donovani* [67,68], consistent with there being high concentrations of the amino acid in the insect vector; however its conversion to glutamate is worth consideration. The related trypanosomatid *Trypanosoma brucei* when grown in proline-enriched medium produced greatly increased levels of glutamate, glutathione and trypanothione [69], consistent with this parasite converting proline to glutamate. Thus proline-glutamate interconversions seem a real possibility. Many of the metabolites of the intermediate pathways were detected in the current study, with some interesting differences in intensity between species. The data together suggest that there are distinct species variations in glutamate-proline metabolism.

Both serine and threonine were consumed in great amounts by all three species (S6 Table), and although *Leishmania* has the genes required to make threonine from aspartate semialdehyde and convert it to serine [68], serine uptake is known to be essential for *L*. *major* [70]. Alanine and glycine were secreted in large amounts (S6 Table, Fig 2B), with there being distinct species-specificity; especially for glycine. Some glycine might be derived from the hydrolysis of hippuric acid, which is completely depleted in the spent growth media (Fig 2A, Table 3). Alanine is a known end-product of glucose metabolism [71] and its release is thought to be a mechanism for countering osmotic stress [71]; it has also been reported that glycine may act similarly [72].

Clear species differences were observed concerning the presence of transamination products of valine, leucine and isoleucine in the spent media (S8 Table). The three species consumed similar amounts of leucine, but released very different amounts of the transamination product 4-methyl-2-oxopentanoic acid—*L*. *major* 10-fold more than *L*. *mexicana*. *Leishmania* use leucine for sterol biosynthesis; 4-methyl-2-oxopentanoic acid is generated in the cytoplasm, further oxidised in the mitochondrion to form hydroxymethylglutaryl-CoA and then directly incorporated into sterols without prior conversion to acetyl-CoA [33,73]. However, the extent of this conversion appeared species-specific. By using ^13^C-labelled leucine it was shown that *L*. *mexicana* and *L*. *major* produced 76–52% of their sterols from leucine whereas the comparative figure for *L*.*donovani* was only 24% [73]. The relatively low level of 4-methyl-2-oxopentanoic acid released by *L*. *mexicana* is consistent with the greater use of the keto acid for sterol production, but such a relationship is not true for the other two species suggesting that leucine is probably deaminated for other purposes too.

The finding of this study that several other deamination products of amino acids are released by the three species, most notably the phenylalanine derivative 3-phenyllactate which was released in large amounts by both *L*. *donovani* and *L*. *major* (S8 Table), suggests that there is a common route for amino acid catabolism which most likely involves broad specificity aminotransferase and hydroxyacid dehydrogenase enzymes. Glutamate, a common product of aminotransferase activity, can be metabolised by glutamate dehydrogenase to generate reducing equivalents and ammonia. The release of ammonia by promastigotes, especially by *L*. *mexicana*, is consistent with a role for the broad specificity aminotransferases in redox balance as previously suggested [53].

Overall our data show markedly different metabolic patterns for *L*. *donovani*, *L*. *mexicana* and *L*. *major* (Tables 2 and 3, Fig 2) and reveal distinct differences in the uptake or release of a variety of metabolites by the three species; the metabolism of the three species is far from identical. Most notably, they use considerably different amounts of some amino acids, especially tryptophan and arginine, and catabolise these to end-products, indole-3-lactate and arginic acid respectively, that are simply released. One limitation of this study is that the analysis was carried out using promastigotes. Unfortunately it is currently not possible to undertake such a comparative analysis with amastigotes, either isolated from animals or cultured axenically. This restriction has also been noted in several other recent publications studies [10,11,12]. We propose that if these differences determined for promastigotes also occur between amastigotes of the three species then they could comprise mechanisms that facilitate the survival of the parasites in their mammalian hosts by modulating the immune responses that the host is able to mount against them. The differing consumption of these amino acids may be central to the specific pathologies that the parasites cause and the different host cells in which the parasites reside and multiple. Thus the data that we have generated with promastigotes can be used to formulate hypotheses concerning mechanisms that could mediate the differing biology of the three species in their mammalian host, the challenge, however, is to show whether or not amastigotes similarly differ. Nevertheless, the outcome of this study provides clear evidence on the value of global metabolomic analyses in detecting species-specific metabolic features, and also give a framework within which to conduct further studies exploring the host-parasite interactions.

## Supporting Information

S1 Fig Fragmentation of arginic acid. (TIF)

S2 Fig Heatmap showing the 50 most abundant metabolites in *L*. *donovani* and comparative levels in the other species. Key to intensities: red, >3 x 10^7^; yellow, >3 x10^6^; blue < 3 x10^5^.
(DOCX)

S3 Fig Heatmap of the 25 metabolites most abundant in *L*. *donovani* spent medium and their levels in the spent media of the other two species and in the original growth medium. Key to intensities: red, >3 x 10^7^; yellow, >3 x10^6^; blue < 3 x10^5^.
(DOCX)

S4 Fig Growth of *Leishmania* species in HOMEM with 10% FCS. *Leishmania* cultures were initiated at 2.5 x 10^5^ cells/ml in 10 ml HOMEM with 10% FCS on (culture day 1) and growth at 26°C was monitored by determining cells densities microscopically every 24 h for 7 days (culture day 8). The data are the means ± standard deviation (SD) from 3 replicate cultures.
(PPTX)

S5 Fig Standard curves of amino acids generated from LC-MS intensity values obtained from varying concentration of the amino acids of interest. (TIF)

S6 Fig Standard curves of tryptophan and its potential catabolic products generated from LC-MS intensity values obtained from varying the concentrations of the compounds of interest. (TIF)

S7 Fig Heatmap showing metabolites accumulating in spent media for the three *Leishmania* species resulting from amino acid deamidation. Key to intensities: red, >3 x 10^7^; yellow, >3 x10^6^; blue < 3 x10^5^.
(DOCX)

S8 Fig The concentrations of selected amino acids determined by LC/MS. Medium A, HOMEM with 10% FCS; medium C, mHOMEM (without essential amino acids) with 10% FCS; 10% FCS in H_2_O; 10% dFCS in H_2_0. The data are means ± SD (n = 3).
(PPTX)

S9 Fig Growth of *L*. *major* with different concentrations of ‘essential’ amino acids. Cultures of *L*. *major* were initiated at 2.5 x 10^5^ cells/ml. Growth media: A, HOMEM with 10% FCS; B, HOMEM with 10% dFCS; C, mHOMEM with 10% FCS; D, mHOMEM with 10% dFCS. The figure shows cell titres in day 3 and day 6 cultures of *L*. *major*. Data are the means ± SD of 3 biological replicates.
(PPTX)

S10 Fig Metabolomic analysis of spent media obtained when *L*. *major* was grown with different concentrations of ‘essential’ amino acids using medium A (HOMEM with 10% FCS) or medium C (mHOMEM with 10% FCS). Cultures of *L*. *major* were initiated at 2.5 x 10^5^ cells/ml and samples taken on days 3 and 6. The data are the amino acid concentrations in spent media samples and in uninfected medium, used as a control. They are means ± SD of 3 biological replicates.
(PPTX)

S11 Fig Calibration curves for amino acids and derivatives. (PPTX)

S12 Fig Comparison of growth of *L*. *major* in media with different concentrations of amino acids. (a) Growth in media with 10% FCS. *L*. *major* promastigotes from a day 6 culture were washed 2-times in PBS and cultures at 2.5 x 10^5^ cells/ml in the following media supplemented with 10% FCS: medium A, HOMEM;—Trp, HOMEM without tryptophan;-Arg medium, HOMEM without arginine; medium C, mHOMEM. Cell titres were determined on days 3 and 6; data are means ± SD of 3 biological replicates. (b) Growth in the same media with 2% FCS.
(PPTX)

S1 Table Standards solutions of metabolites used to aid metabolite identification. (XLSX)

S2 Table Metabolites of confirmed identity detected in the extract samples and/or spent media. The colour coding is explained at the end of the table.
(XLSX)

S3 Table Formulae of HOMEM and HOMEM without amino acids and amino acid concentrations in HOMEM and mHOMEM (modified HOMEM without essential amino acids). (XLSX)

S4 Table Metabolites identified in extracts of promastigotes of the three *Leishmania* species. The colour coding is explained at the end of the table.
(XLSX)

S5 Table Metabolites identified in the media from cultures of promastigotes of the three *Leishmania* species. The colour coding is explained at the end of the table.
(XLSX)

S6 Table Concentrations of amino acids and derivatives in the media, as analysed using the standard curves shown in S5 Fig. (XLSX)

S7 Table Relative levels of arginine metabolites detected in the cell extracts and spent media. (XLSX)

S8 Table Concentrations of catabolites of phenylalanine, tyrosine, leucine, isoleucine and valine in the media, as analysed using standard curves. (XLSX)
