# Supplementary figures and images for: Metabolomic Analyses of Leishmania Reveal Multiple Species Differences and Large Differences in Amino Acid Metabolism
Source: PLoS One. 2015 Sep 14;10(9):e0136891. doi: 10.1371/journal.pone.0136891 (PMC4569581; doi:10.1371/journal.pone.0136891)

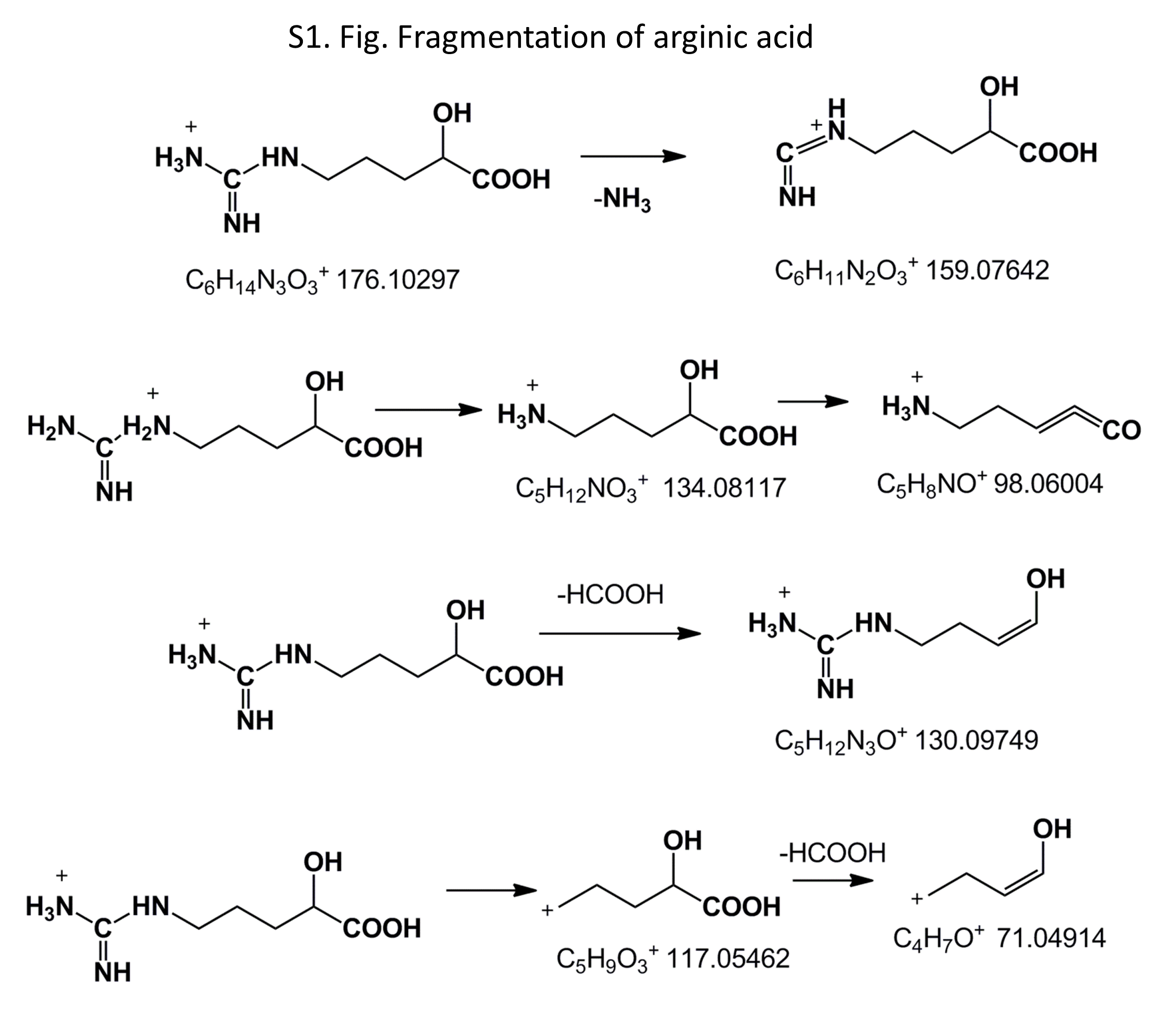

Supplement: S1 Fig — (TIF) [file pone.0136891.s001.TIF]

## Slide 1
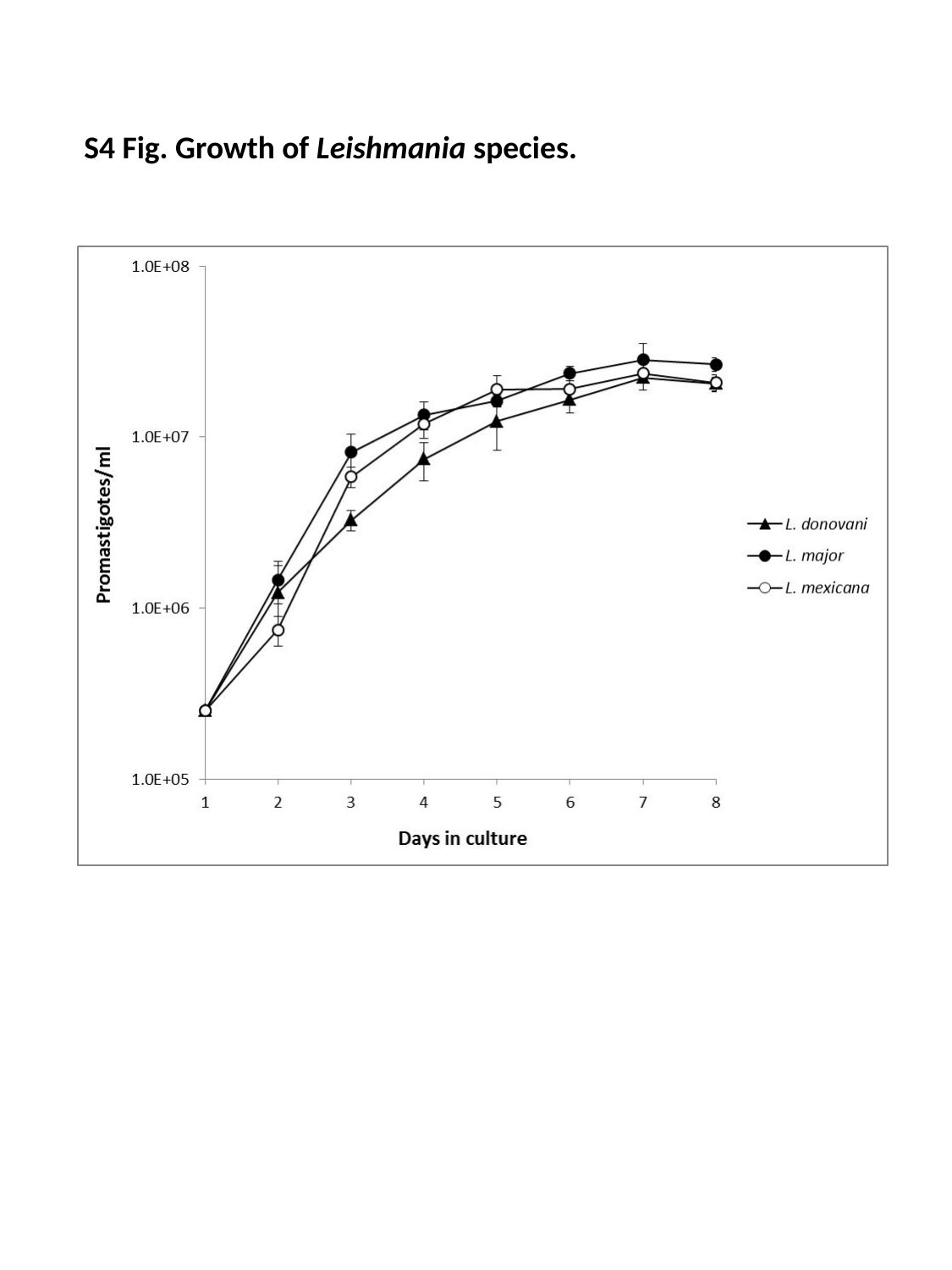

S4 Fig. Growth of Leishmania species.

Supplement: S4 Fig — Leishmania cultures were initiated at 2.5 x 10^5^ cells/ml in 10 ml HOMEM with 10% FCS on (culture day 1) and growth at 26°C was monitored by determining cells densities microscopically every 24 h for 7 days (culture day 8). The data are the means ± standard deviation (SD) from 3 replicate cultures. (PPTX) [file pone.0136891.s004.pptx]

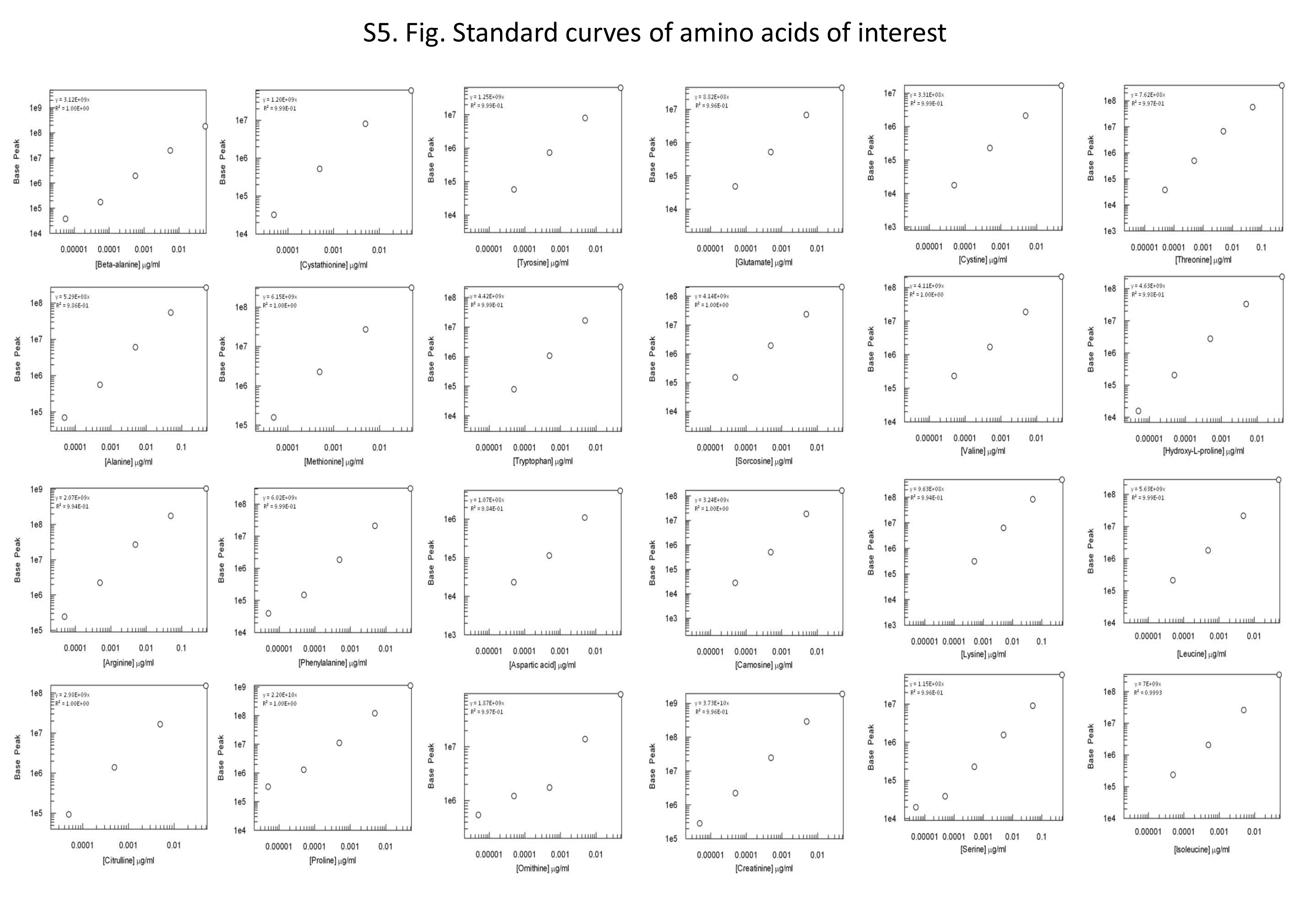

Supplement: S5 Fig — (TIF) [file pone.0136891.s005.TIF]

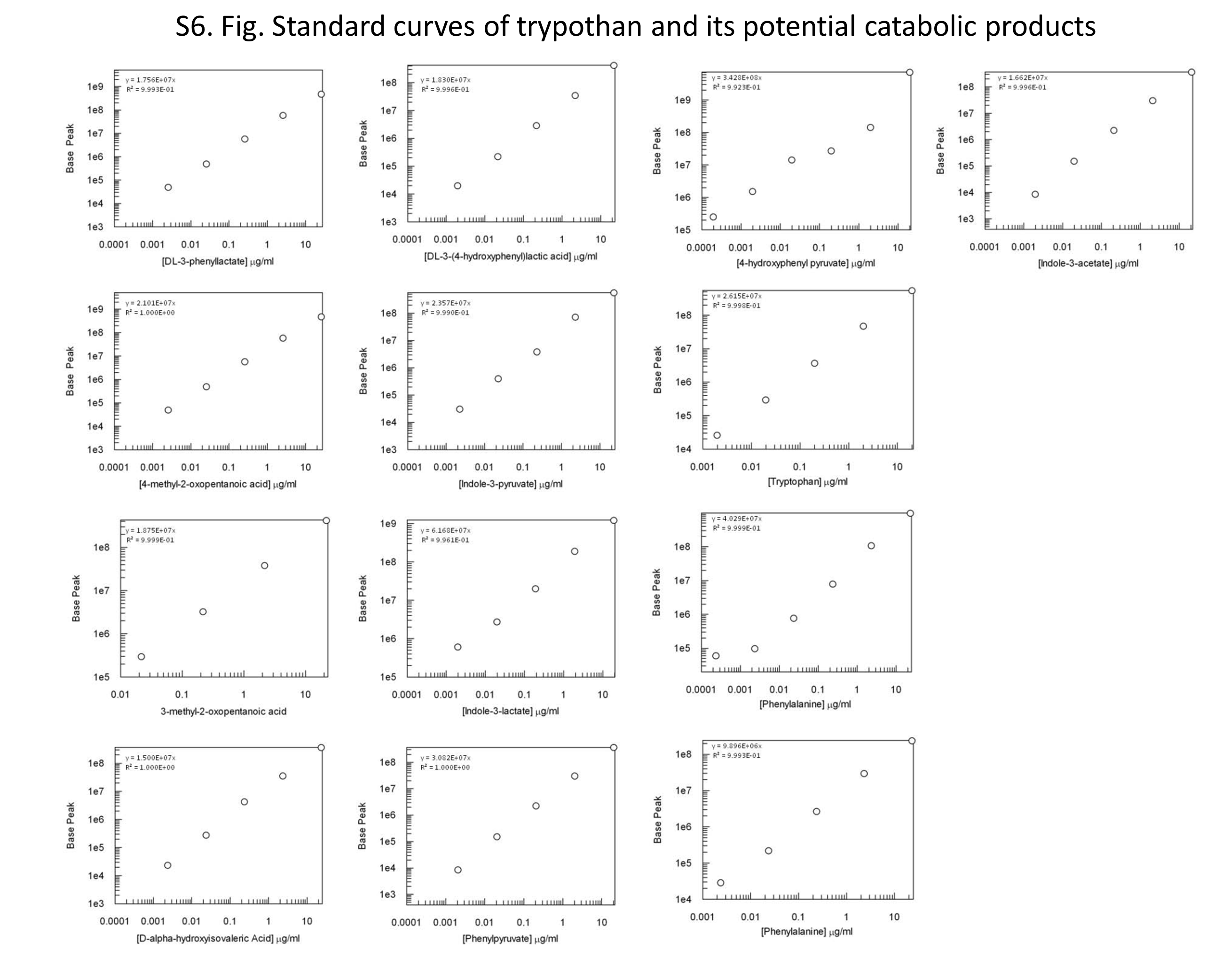

Supplement: S6 Fig — (TIF) [file pone.0136891.s006.TIF]

## Slide 1
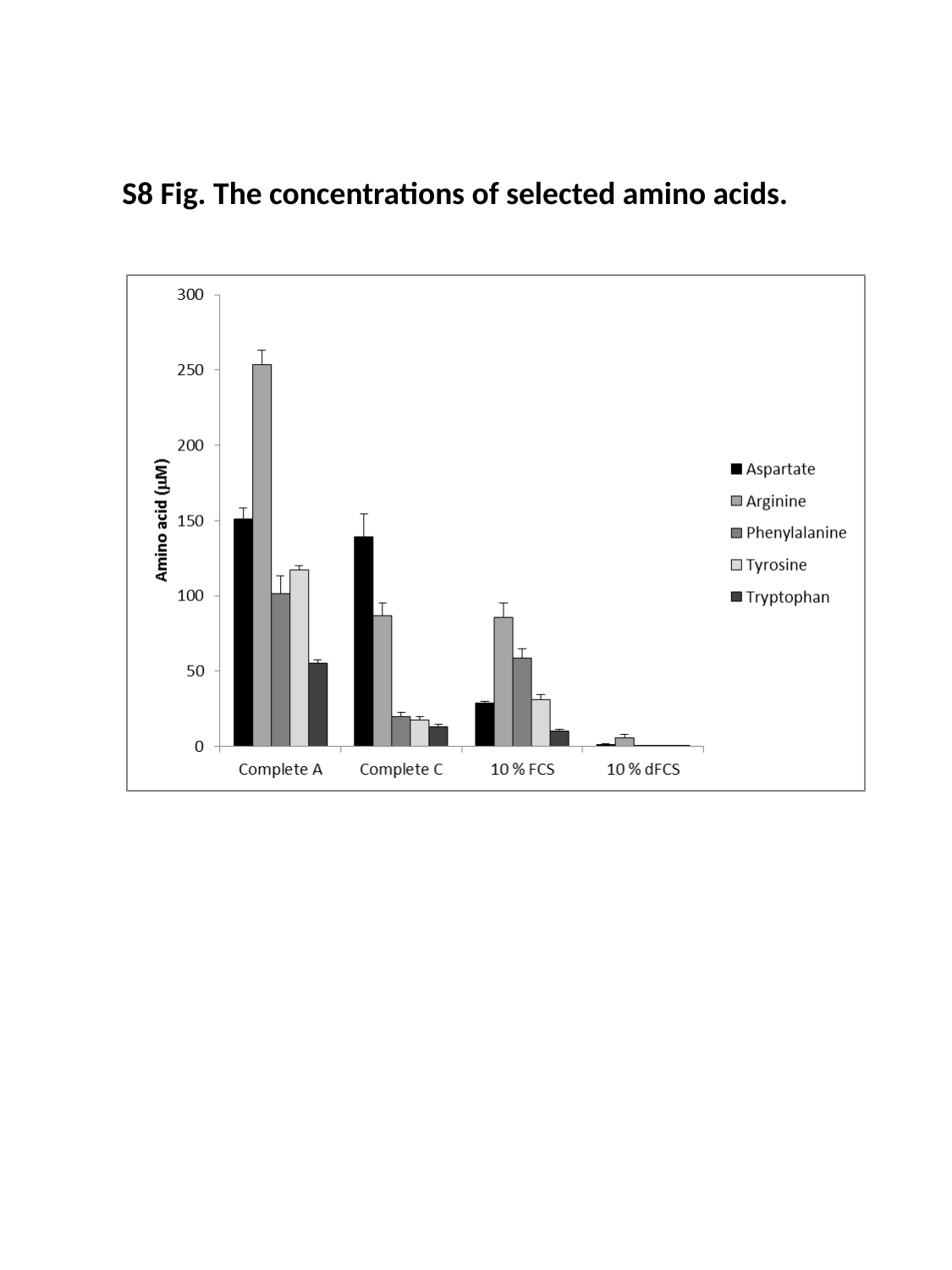

S8 Fig. The concentrations of selected amino acids.

Supplement: S8 Fig — Medium A, HOMEM with 10% FCS; medium C, mHOMEM (without essential amino acids) with 10% FCS; 10% FCS in H2O; 10% dFCS in H20. The data are means ± SD (n = 3). (PPTX) [file pone.0136891.s008.pptx]

## Slide 1
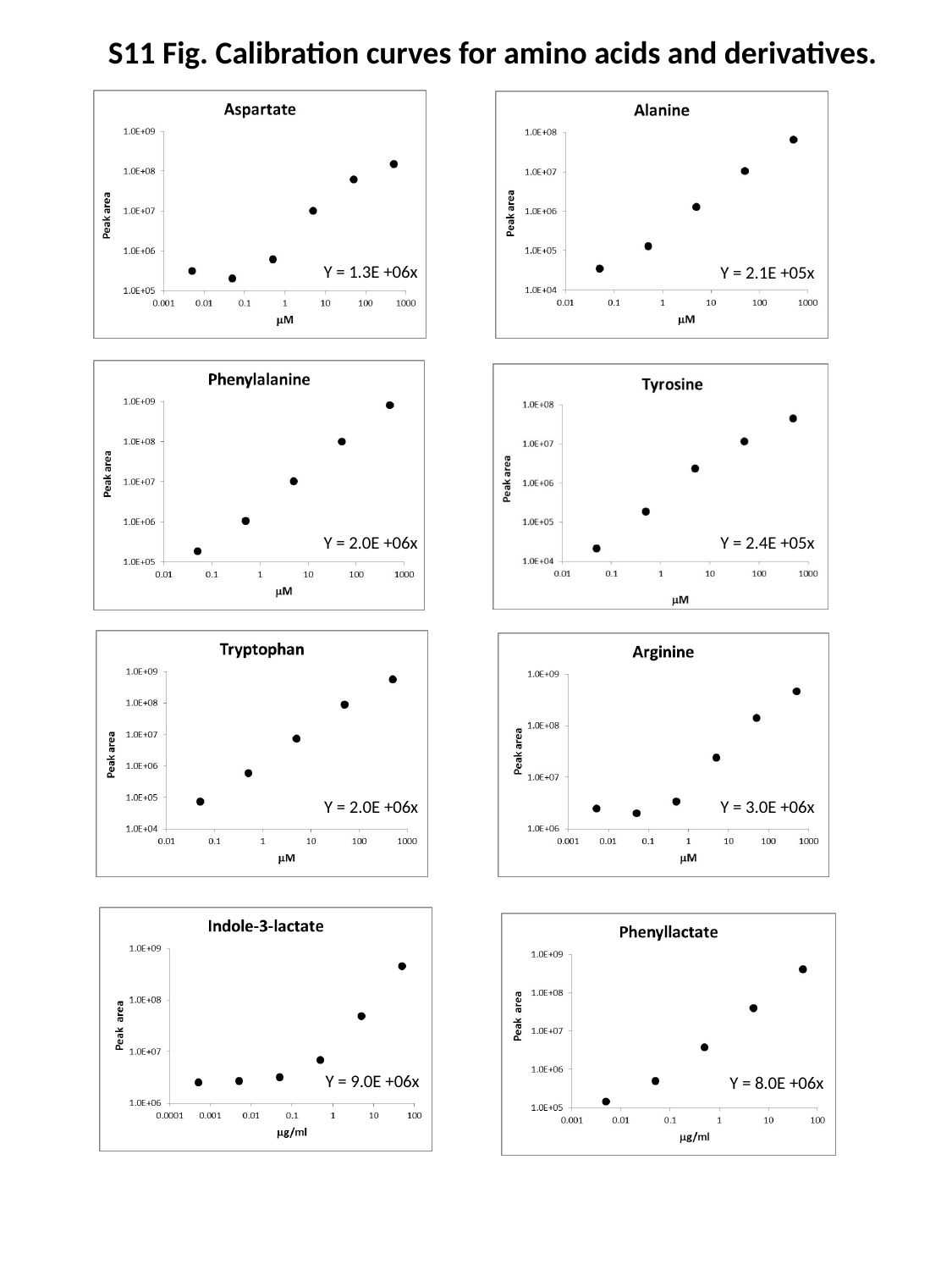

S11 Fig. Calibration curves for amino acids and derivatives.
Y = 1.3E +06x
Y = 2.1E +05x
Y = 2.4E +05x
Y = 2.0E +06x
Y = 2.0E +06x
Y = 3.0E +06x
Y = 9.0E +06x
Y = 8.0E +06x

Supplement: S11 Fig — (PPTX) [file pone.0136891.s011.pptx]
