# Supplementary material for: Metabolomic Analyses of Leishmania Reveal Multiple Species Differences and Large Differences in Amino Acid Metabolism
Source: PLoS One. 2015 Sep 14;10(9):e0136891. doi: 10.1371/journal.pone.0136891 (PMC4569581; doi:10.1371/journal.pone.0136891)
Supplement: S2 Fig — Key to intensities: red, >3 x 10^7^; yellow, >3 x10^6^; blue < 3 x10^5^. (DOCX) [file pone.0136891.s002.docx]

**S2 Fig. The 50 most abundant metabolites in *L. donovani* extracts.**

| **Metabolite** | *L. donovani* | *L. major* | *L. mexicana* |
| --- | --- | --- | --- |
| Arginic acid |  |  |  |
| Imidazole lactate |  |  |  |
| beta-Alanine |  |  |  |
| L-Glutamate |  |  |  |
| L-Glutamine |  |  |  |
| Orthophosphate |  |  |  |
| Valine |  |  |  |
| IsoLeucine |  |  |  |
| Leucine |  |  |  |
| Pipecolate |  |  |  |
| Succinate |  |  |  |
| (Seryl)adenylate |  |  |  |
| (S)-Malate |  |  |  |
| 3-(3-Hydroxy-phenyl)-propanoic acid |  |  |  |
| Ovothiol A |  |  |  |
| Threonine |  |  |  |
| Gulonate |  |  |  |
| Aspartate |  |  |  |
| Ovothiol A disulfide |  |  |  |
| Choline |  |  |  |
| Sorbitol |  |  |  |
| Deoxyribose |  |  |  |
| Citrate |  |  |  |
| 3-(4-Hydroxyphenyl)lactate |  |  |  |
| Phenyllactic acid |  |  |  |
| L-Cystathionine |  |  |  |
| Betaine |  |  |  |
| Imidazole-4-acetaldehyde |  |  |  |
| Asparagine |  |  |  |
| Glycine |  |  |  |
| Hydroxyproline |  |  |  |
| AMP |  |  |  |
| ADP |  |  |  |
| Arginine |  |  |  |
| Phenylalanine |  |  |  |
| Lysine |  |  |  |
| 2-Phospho-D-glycerate |  |  |  |
| Histidine |  |  |  |
| Methionine |  |  |  |
| Serine |  |  |  |
| Indolelactate |  |  |  |
| ATP |  |  |  |
| 2-Oxoglutarate |  |  |  |
| 5-Acetamidopentanoate |  |  |  |
| D-Glycerate |  |  |  |
| Phosphite |  |  |  |
| Adenosine |  |  |  |
| Trypanothione disulfide |  |  |  |
| UDP-glucose |  |  |  |
| Sulfate |  |  |  |
| UMP |  |  |  |

Key to intensities: red, >3 x 10^7^; yellow, >3 x 10^6^ ; blue, <3 x 10^5^
