# Supplementary material for: Metabolomic Analyses of Leishmania Reveal Multiple Species Differences and Large Differences in Amino Acid Metabolism
Source: PLoS One. 2015 Sep 14;10(9):e0136891. doi: 10.1371/journal.pone.0136891 (PMC4569581; doi:10.1371/journal.pone.0136891)
Supplement: S3 Fig — Key to intensities: red, >3 x 10^7^; yellow, >3 x10^6^; blue < 3 x10^5^. (DOCX) [file pone.0136891.s003.docx]

**S3 Fig. Heatmap of the 25 metabolites most abundant in *L. donovani* spent medium and their relative levels on the spent media of the other two species.**

| **Metabolite** | medium | *L. donovani* | *L. major* | *L. mexicana* |
| --- | --- | --- | --- | --- |
| Succinate |  |  |  |  |
| Isoleucine |  |  |  |  |
| Valine |  |  |  |  |
| Orthophosphate |  |  |  |  |
| Arginine |  |  |  |  |
| Glutamine |  |  |  |  |
| Histidine |  |  |  |  |
| Phenylalanine |  |  |  |  |
| Glucose |  |  |  |  |
| Choline |  |  |  |  |
| Creatinine |  |  |  |  |
| Pyruvate |  |  |  |  |
| Creatine |  |  |  |  |
| Arginic acid |  |  |  |  |
| Citrate |  |  |  |  |
| Hydroxyphenyllactate |  |  |  |  |
| Malate |  |  |  |  |
| 3-Methyl-2-oxopentanoic acid |  |  |  |  |
| beta-Alanine |  |  |  |  |
| Tyrosine |  |  |  |  |
| Methionine S-oxide |  |  |  |  |
| Indolelactate |  |  |  |  |
| Threonine |  |  |  |  |
| Glutamate |  |  |  |  |
| Leucine |  |  |  |  |

Key to intensities: red, >3 x 10^7^; yellow, >3 x10^6^; blue <3 x10^5^
