# Supplementary material for: Metabolomic Analyses of Leishmania Reveal Multiple Species Differences and Large Differences in Amino Acid Metabolism
Source: PLoS One. 2015 Sep 14;10(9):e0136891. doi: 10.1371/journal.pone.0136891 (PMC4569581; doi:10.1371/journal.pone.0136891)
Supplement: S7 Fig — Key to intensities: red, >3 x 10^7^; yellow, >3 x10^6^; blue < 3 x10^5^. (DOCX) [file pone.0136891.s007.docx]

**S7 Fig.** Metabolites accumulating in spent media for the three *Leishmania* species resulting from amino acid deamidation.

| **Metabolite** | Medium | *L. donovani* | *L. major* | *L. mexicana* |
| --- | --- | --- | --- | --- |
| Imidazole oxopropanoate |  |  |  |  |
| arginic acid |  |  |  |  |
| 3-(4-Hydroxyphenyl)lactate |  |  |  |  |
| (S)-3-Methyl-2-oxopentanoic acid |  |  |  |  |
| Indolelactate |  |  |  |  |
| 3-(4-Hydroxyphenyl)pyruvate |  |  |  |  |
| Phenylpyruvate |  |  |  |  |
| Imidazole-4-acetaldehyde |  |  |  |  |
| 2-Oxoglutarate |  |  |  |  |
| Hydroxyglutarate |  |  |  |  |
| Acetolactate |  |  |  |  |
| indole-pyruvate |  |  |  |  |
| 2-oxobutenoate |  |  |  |  |
| 5-Guanidino-2-oxopentanoate |  |  |  |  |
| 3-Methyl-2-oxobutanoic acid |  |  |  |  |
| Imidazol-5-yl-pyruvate |  |  |  |  |
| Imidazole-4-acetate |  |  |  |  |
| Phenylpyruvate |  |  |  |  |
| 2-oxobut-3-enanoate |  |  |  |  |
| Imidazole-4-methanol |  |  |  |  |
| Oxoglutaramate |  |  |  |  |
| Oxoglutaramate isomer |  |  |  |  |
| 3-Mercaptolactate |  |  |  |  |
| mercapto hydroxybutyric acid |  |  |  |  |
| mercaptohydroxybutyric acid isomer |  |  |  |  |
| 3-(3,4-Dihydroxyphenyl)pyruvate |  |  |  |  |
| L-Homocitrulline |  |  |  |  |
| Indole-3-acetate |  |  |  |  |

Key to intensities: red, >3 x 10^7^; yellow, >3 x 10^6^ ; blue, <3 x 10^5^
