# Supplementary material for: Metabolomic Analyses of Leishmania Reveal Multiple Species Differences and Large Differences in Amino Acid Metabolism
Source: PLoS One. 2015 Sep 14;10(9):e0136891. doi: 10.1371/journal.pone.0136891 (PMC4569581; doi:10.1371/journal.pone.0136891)
Supplement: S9 Fig — Cultures of L. major were initiated at 2.5 x 10^5^ cells/ml. Growth media: A, HOMEM with 10% FCS; B, HOMEM with 10% dFCS; C, mHOMEM with 10% FCS; D, mHOMEM with 10% dFCS. The figure shows cell titres in day 3 and day 6 cultures of L. major. Data are the means ± SD of 3 biological replicates. (PPTX) [file pone.0136891.s009.pptx]

## Slide 1
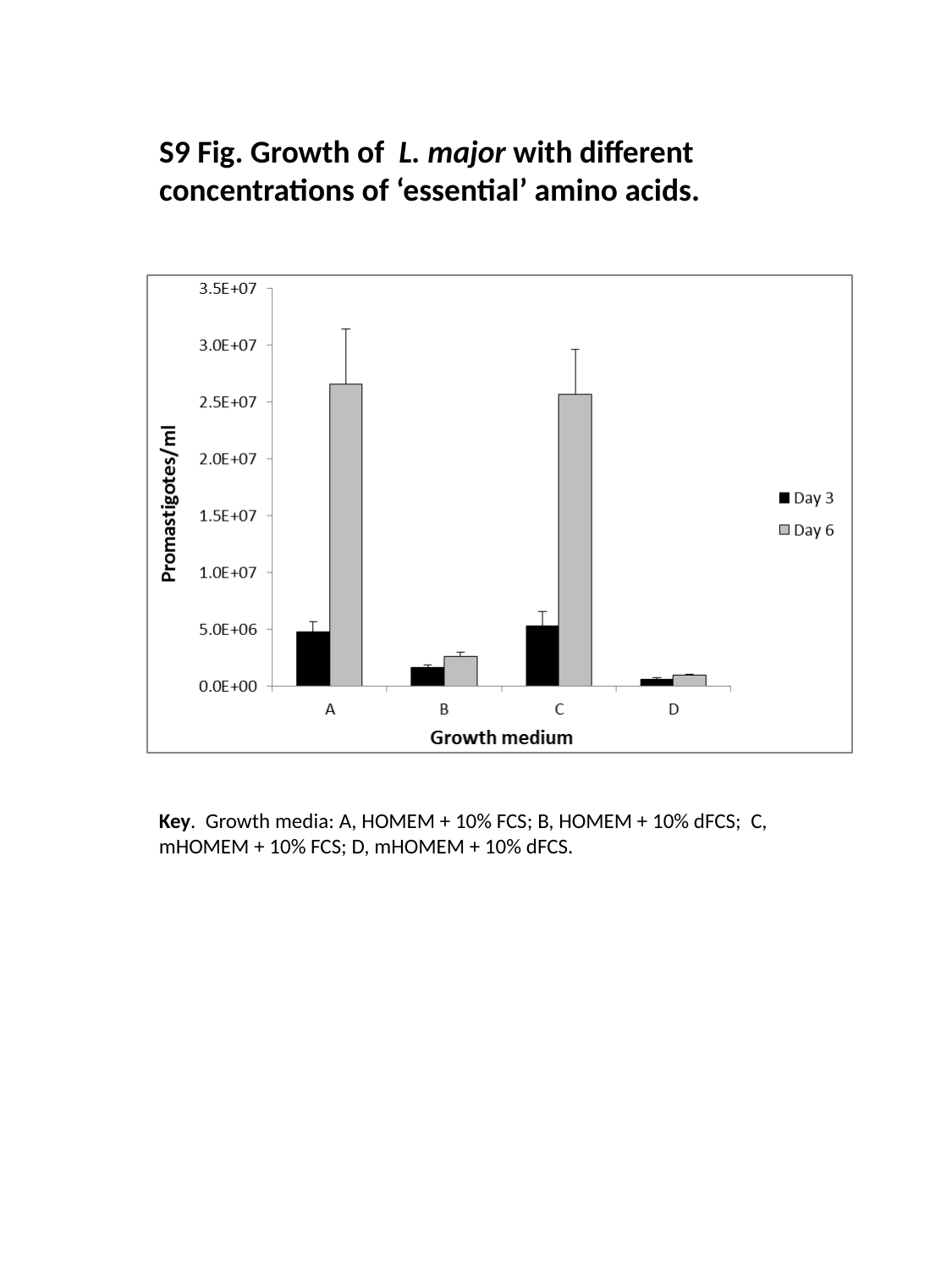

S9 Fig. Growth of L. major with different concentrations of ‘essential’ amino acids.
Key. Growth media: A, HOMEM + 10% FCS; B, HOMEM + 10% dFCS; C, mHOMEM + 10% FCS; D, mHOMEM + 10% dFCS.
