# Supplementary material for: Metabolomic Analyses of Leishmania Reveal Multiple Species Differences and Large Differences in Amino Acid Metabolism
Source: PLoS One. 2015 Sep 14;10(9):e0136891. doi: 10.1371/journal.pone.0136891 (PMC4569581; doi:10.1371/journal.pone.0136891)
Supplement: S10 Fig — Cultures of L. major were initiated at 2.5 x 10^5^ cells/ml and samples taken on days 3 and 6. The data are the amino acid concentrations in spent media samples and in uninfected medium, used as a control. They are means ± SD of 3 biological replicates. (PPTX) [file pone.0136891.s010.pptx]

## Slide 1
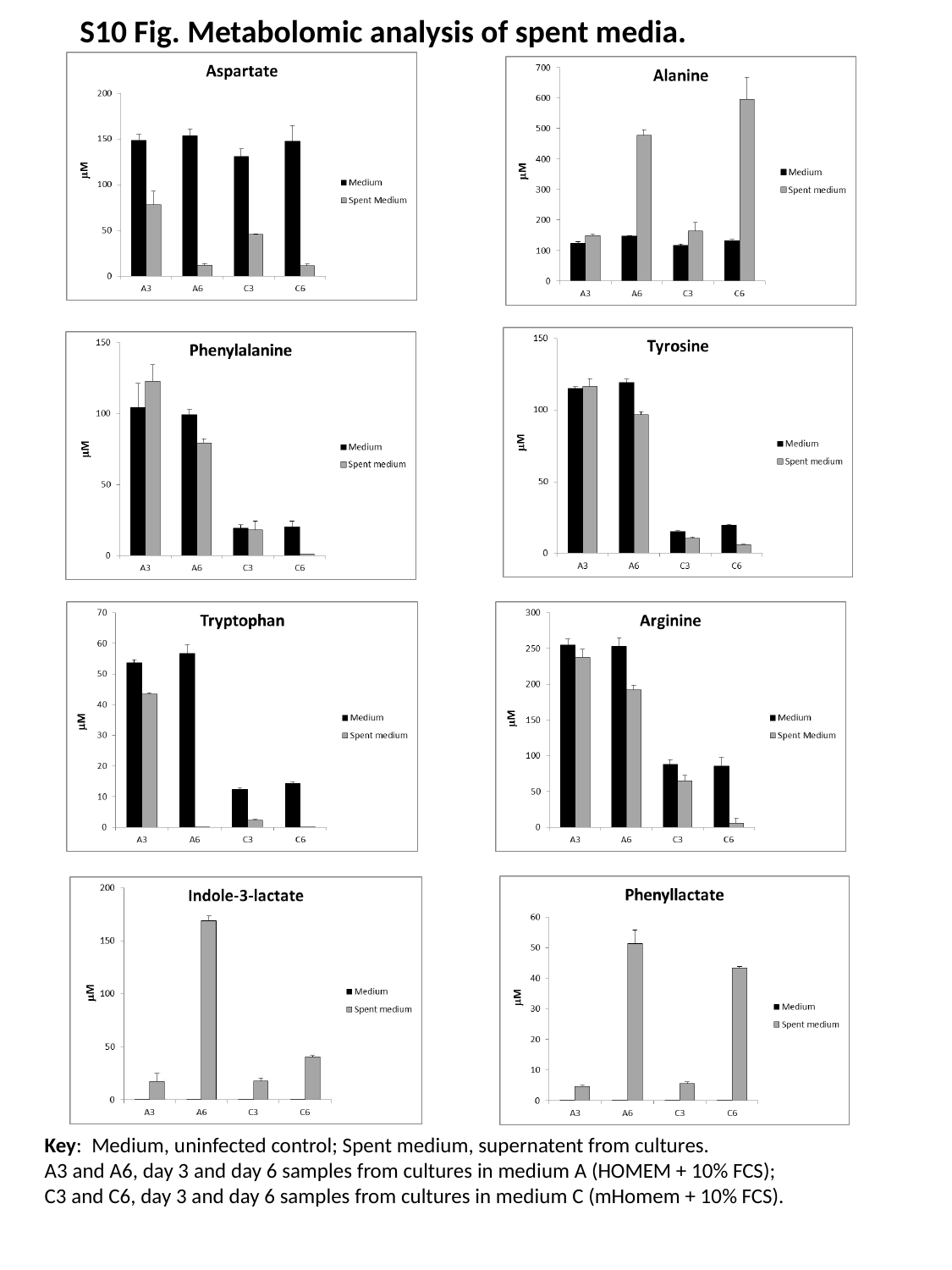

S10 Fig. Metabolomic analysis of spent media.
Key: Medium, uninfected control; Spent medium, supernatent from cultures.
A3 and A6, day 3 and day 6 samples from cultures in medium A (HOMEM + 10% FCS);
C3 and C6, day 3 and day 6 samples from cultures in medium C (mHomem + 10% FCS).
