# Supplementary material for: Metabolomic Analyses of Leishmania Reveal Multiple Species Differences and Large Differences in Amino Acid Metabolism
Source: PLoS One. 2015 Sep 14;10(9):e0136891. doi: 10.1371/journal.pone.0136891 (PMC4569581; doi:10.1371/journal.pone.0136891)
Supplement: S12 Fig — (a) Growth in media with 10% FCS. L. major promastigotes from a day 6 culture were washed 2-times in PBS and cultures at 2.5 x 10^5^ cells/ml in the following media supplemented with 10% FCS: medium A, HOMEM;—Trp, HOMEM without tryptophan;-Arg medium, HOMEM without arginine; medium C, mHOMEM. Cell titres were determined on days 3 and 6; data are means ± SD of 3 biological replicates. (b) Growth in the same media with 2% FCS. (PPTX) [file pone.0136891.s012.pptx]

## Slide 1
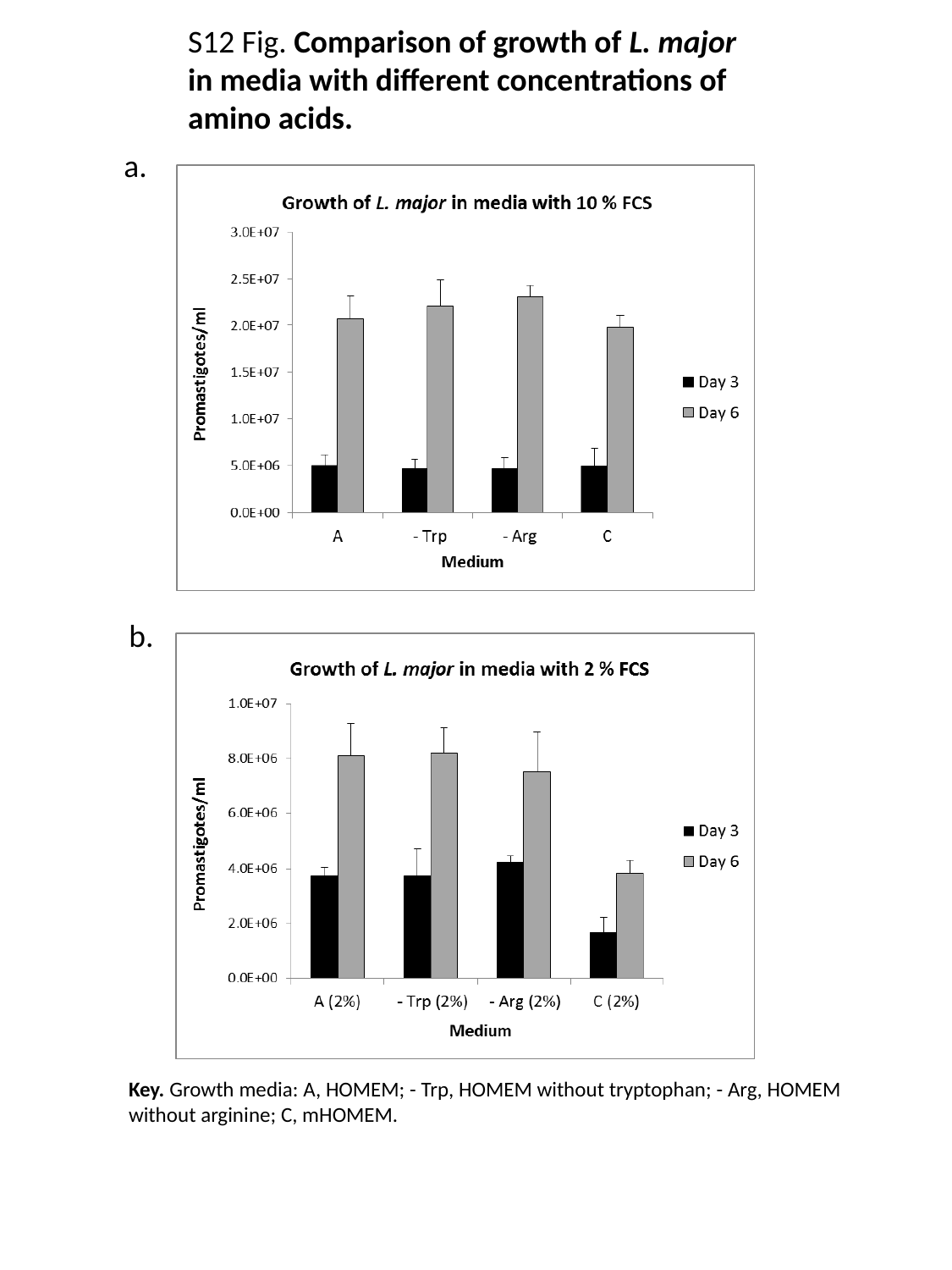

S12 Fig. Comparison of growth of L. major in media with different concentrations of amino acids.
a.
b.
Key. Growth media: A, HOMEM; - Trp, HOMEM without tryptophan; - Arg, HOMEM without arginine; C, mHOMEM.
